# An Optimized Conversion of Spermatogonial Stem Cells Into Spinal Cord Neurons Enhances Functional Recovery in Rats After Spinal Cord Injury

**DOI:** 10.1002/cns.70844

**Published:** 2026-04-15

**Authors:** Xinyu Guo, Yongjie Zhang, Haihong Zhang, Hao Yang

**Affiliations:** ^1^ Department of Surgery The Second Hospital of Xi'an Medical University Xi'an China; ^2^ Department of Electromyography Hong Hui Hospital, Xi'an Jiaotong University Xi'an China; ^3^ Department of Orthopedics The Second Hospital of Lanzhou University Lanzhou China; ^4^ Translational Medicine Center Hong Hui Hospital, Xi'an Jiaotong University Xi'an China

**Keywords:** cell differentiation, JAK/STAT signaling pathway, spermatogonial stem cells, spinal cord injury, stem cell therapy

## Abstract

**Background:**

The inflammatory response following spinal cord injury (SCI) is a critical factor contributing to neural dysfunction. This response exacerbates neural damage in the injured area by facilitating the infiltration of inflammatory cells and the release of cytokines, thereby remarkably impeding neural repair and functional recovery. Spermatogonial stem cells (SSCs) have the potential for spinal cord nerve regeneration. Concurrently, olfactory ensheathing cells (OECs), a unique cell type with neurorepair potential, can effectively enhance nerve regeneration through the secretion of various neurotrophic factors, the suppression of inflammatory responses, and improvements in the injury microenvironment. However, research concerning OEC induction systems and the differentiation of SSCs into spinal cord neurons remains limited. This study aimed to investigate the synergistic effects of OECs and SSCs to develop a more effective and safe stem cell therapy strategy for SCI.

**Methods:**

This study integrates in vitro and in vivo methodologies to investigate the mechanisms through which an efficient induction system facilitates the differentiation of SSCs into neurons. Initially, primary SSCs were cultured and characterized through immunofluorescence, and the molecular mechanisms underlying the induction system were examined via western blot and quantitative reverse transcription (qRT–PCR) methods. An inflammatory environment was subsequently developed in a lipopolysaccharide (LPS)‐activated microglial model to evaluate the impact of the induction system on SSC differentiation. Finally, differentiated SSCs were transplanted into a traumatic SCI model, and functional recovery was assessed via the Basso, Beattie and Bresnahan (BBB) locomotor scale, as well as electrophysiology, including somatosensory evoked potential (SEP) and motor evoked potential (MEP) tests.

**Results:**

In vitro experimental results demonstrated that the activation of the OEC induction system significantly enhanced the efficiency of the differentiation of SSCs into spinal cord neurons. Notably, in the inflammatory milieu, an optimal concentration of lipopolysaccharide (LPS) significantly augments both neuronal differentiation efficiency and survival rates. In addition, compared with those in the other experimental groups, the induced neuronal‐like cells exhibited genuine neuronal characteristics. Importantly, JAK2/STAT3 pathway signaling plays a crucial role in this intricate cellular transformation. Suppression of the JAK2/STAT3 signaling pathway significantly improved differentiation efficiency and facilitated neural repair. In vivo experimental results revealed that the transplantation of differentiated spinal neurons into the lesioned spinal cord substantially improved sensory and motor functions, as evidenced by behavioral assessments (such as BBB scoring) and electrophysiological tests (MEP and SEP).

**Conclusion:**

This study developed an efficient induction system that facilitates the differentiation of SSCs into spinal cord neurons. The system also significantly improved neuronal differentiation and survival under LPS‐induced inflammatory conditions. Crucially, the JAK2/STAT3 signaling pathway has been identified as a critical regulatory mechanism in this complex process. Intriguingly, inhibition of the JAK2/STAT3 pathway significantly increases neuronal differentiation efficiency. The transplantation of these differentiated cells resulted in the functional recovery of sensory and motor functions in the lesioned spinal cord. These findings provide a novel therapeutic strategy for stem cell‐based treatment of spinal cord injuries and lay both a theoretical and practical foundation for clinical applications.

## Graphical abstract

The OEC induction system significantly enhanced SSC differentiation into spinal neurons, with optimal LPS concentration improving outcomes. Induced cells exhibited neuronal traits, relying on the JAK2/STAT3 pathway, whose suppression aided differentiation and repair. Transplanting differentiated neurons into injured cords improved functions, validated by tests. This study offers an effective SSC differentiation method, emphasizes the JAK2/STAT3 pathway's importance, and proposes a new spinal cord injury treatment strategy.

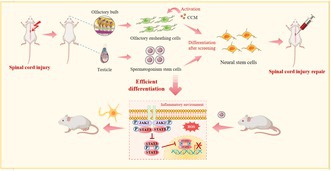

## 1 Introduction

Spinal cord injury (SCI) is a particularly devastating disease of the central nervous system (CNS) caused by either traumatic or nontraumatic mechanical insults and is typically characterized by sensorimotor function deficits due to the degeneration or loss of spinal cord neurons. Following SCI, the body initiates a series of complex pathophysiological responses. Among these mechanisms, the inflammatory response is a critical mechanism that exacerbates the severity of SCI and contributes to the development of numerous sequelae, primarily through the induction of neural cell apoptosis in the affected area and the exacerbation of secondary injury [1]. During the acute phase of SCI, there is a marked infiltration of inflammatory cells, including neutrophils, macrophages, and activated microglia, at the injury site. These inflammatory cells release a variety of cytokines and chemotactic factors, disrupting the homeostasis of the local environment. This disruption results in degradation of the extracellular matrix, compromise of the blood–brain barrier, and further neuronal necrosis [2]. Therefore, effective modulation of the complex inflammatory response, alleviation of secondary injury, and restoration of damaged neurons are crucial for mitigating the adverse outcomes associated with SCI.

With the ongoing advancements in clinical translational medicine and stem cell therapy research, stem cells have demonstrated tremendous potential for application in the repair of tissue damage resulting from cell loss. Spermatogonial stem cells (SSCs), a distinct category of adult stem cells located on the basement membrane of seminiferous tubules, possess self‐renewal capabilities and can differentiate into spermatogonia, which subsequently undergo meiosis to produce sperm. Compared with embryonic stem cells (ESCs), induced pluripotent stem cells (iPSCs), and mesenchymal stem cells (MSCs), SSCs have advantages such as ease of accessibility and the absence of ethical controversies and tumorigenicity [3, 4, 5]. Consequently, their potential for clinical application is considerable. Research has demonstrated that SSCs can be efficiently induced into neurons through straightforward procedures without complex genetic modifications [6, 7, 8]. In recent years, the rapid progress in regenerative medicine has highlighted the cellular characteristics of SSCs in multiple research fields. In addition to their conventional reproductive functions, SSCs also exhibit pluripotency with the capacity to differentiate into hepatocytes, cardiomyocytes, neurons, osteoblasts, pancreatic islet cells, and prostate epithelial cells under specific conditions [9, 10, 11]. These findings provide novel insights into the potential clinical applications of SSCs, particularly in the treatment of neural injuries and neurodegenerative diseases. Despite the promising potential of SSCs in regenerative medicine, several challenges persist in their clinical applications, particularly in the treatment of SCI. Research indicates that after transplantation into the injured spinal cord, transplanted cells often fail to achieve long‐term survival, with only a minimal number of cells persisting, as the majority undergo necrosis shortly after transplantation, thereby failing to integrate effectively into the damaged neural network [12, 13, 14]. Furthermore, the microenvironment following SCI progressively deteriorates, severely hindering the regeneration of damaged neurons and compromising the survival of transplanted cells. This compromised microenvironment is characterized by degenerated neurons, reactive astrocytes, disrupted axonal myelin sheaths, and microglia, along with the release of numerous inhibitory growth factors.

To address the inflammatory milieu following SCI and facilitate neural repair, OECs have emerged as a highly promising cell type and have attracted considerable scholarly attention in recent years. OECs are a unique type of glial cell with the capacity to enhance nerve regeneration and repair. Recent compelling evidence suggests that OECs not only survive at the site of SCI but also promote axonal regeneration and myelin reconstruction [15]. In addition, OECs secrete a variety of neurotrophic factors, including brain‐derived neurotrophic factor (BDNF), glial cell‐derived neurotrophic factor (GDNF), and neurotrophin‐3 (NT‐3), which activate specific signaling pathways to support neuronal survival, proliferation, and axonal growth. Within the context of the post‐SCI inflammatory environment, these factors play crucial roles in inhibiting the excessive activation of inflammatory cells and thus mitigating secondary injury [16]. Additionally, further investigations revealed that, compared with their nonactivated counterparts, curcumin‐activated OECs exhibit significantly enhanced cytokine secretion capabilities. Curcumin, a natural anti‐inflammatory and antioxidant compound, has been shown to inhibit the NF‐κB signaling pathway, thereby reducing the release of inflammatory cytokines and fostering a more favorable microenvironment for OECs to increase neuronal survival and facilitate functional recovery [17]. As a result, the potential of curcumin as an adjunctive therapeutic agent for the treatment of SCI merits further investigation. In the inflammatory milieu characteristic of SCI, the transplantation of stem cells, such as SSCs, frequently encounters challenges related to low differentiation efficiency and poor cell survival rates [18]. The oxidative stress products and inflammatory mediators released by inflammatory cells can impede stem cell proliferation and differentiation and induce apoptosis. Nonetheless, research indicates that cotransplantation of stem cells with activated OECs significantly modifies this scenario. Activated OECs substantially improve the differentiation efficiency of stem cells within inflammatory environments, facilitate the differentiation of SSCs into spinal cord neurons, and reduce the rate of stem cell apoptosis [19]. In light of these findings, our objective was to optimize the efficient differentiation of SSCs into spinal cord neurons through the involvement of activated OECs. Furthermore, we elucidated the exact molecular mechanisms underlying the conversion of SSCs into spinal cord neurons for the treatment of SCI [20].

In this study, we developed an efficient induction system for the differentiation of SSCs into spinal cord neurons and further investigated the potential of cotransplanting SSCs and activated OECs for the treatment of SCI. Given that the inflammatory microenvironment following SCI, particularly the responses elicited by inflammatory cytokines and immune cells, significantly hinders the survival and integration of transplanted cells, especially within the context of stem cell applications, this study also investigated the role of activated OECs in promoting the differentiation of SSCs into spinal cord neurons and their neuroprotective functions in inflammatory environments. In addition, this study evaluated whether the cotransplantation of SSCs with activated OECs can increase the efficiency of the differentiation of SSCs into spinal cord neurons and improve their survival rate in inflammatory environments. The core objective of this study is to explore the potential and direct intercellular communication between OEC and SSC, rather than completely replicating the in vivo injury environment. Establishing this simplified co‐culture system is a necessary prerequisite and foundation for analyzing specific bidirectional interactions, which provide crucial initial mechanistic insights into a more complex in vivo environment. Consequently, we anticipate that this study will provide a novel approach to address the challenges of cell survival and integration in stem cell therapy for SCI treatment.

## 2 Materials and Methods

### 2.1 Ethical Statement

All animal experiments were conducted in accordance with the guidelines for animal research and use at Xi'an Jiaotong University.

### 2.2 Main Reagents

DMEM/F12 medium (Gibco), fetal bovine serum (FBS) (Gibco), 0.25% EDTA–trypsin (Gibco), DMEM (Gibco), and penicillin/streptomycin solution (Gibco) were purchased from Gibco (USA); TRIzol (Trizol reagent, Invitrogen, USA) was obtained from Invitrogen (USA); lipopolysaccharide (LPS) (SigmaAldrich (#L4524)), complete culture medium (CCM) (Sigma‐Aldrich (St Louis, MO, USA)), poly‐L‐lysine (PLL) (Sigma, Saint Louis, MO), dimethyl sulfoxide (DMSO) (Thermo Fisher Scientific, Massachusetts, USA), retinoic acid (RAR2625, Sigma), β‐mercaptoethanol (β‐ME) (63689, Sigma), isobutylmethylxanthine (IBMX) (I5879, Sigma), DNase I (LS003119, Sigma), dispase II (D4693, Sigma), hyaluronidase (H3384, Sigma), collagenase IV (C5138 Sigma), WP1066 (573097 Sigma), phosphate‐buffered saline (PBS) (P4417), Fluo‐4 AM (F14201; Invitrogen, USA), bovine serum albumin (BSA) (126626 Sigma), and 0.2% gelatin (G1890) were sourced from Sigma–Aldrich (USA); glial cell‐derived neurotrophic factor (GDNF) (450–51), epidermal growth factor (EGF) (400–25), basic fibroblast growth factor (bFGF) (400–29), and leukemia inhibitory factor (LIF) (250–02) were obtained from PEPROTECH (USA); anti‐UCHL1 antibody (K003243P) and anti‐GPR125 antibody (K010000P) were purchased from Solarbio (China); and anti‐p75 antibody (N3908 Sigma), anti‐Tuj1 antibody (12463), anti‐Map2 antibody (8707), anti‐Jak2 antibody (3230), anti‐phospho‐Jak2 antibody (3771), anti‐Stat3 antibody (9139), and anti‐phospho‐Stat3 antibody (9145) were obtained from Cell Signaling Technology.

### 2.3 Isolation and Culture of Spermatogonial Stem Cells

Primary spermatogonial stem cells (SSCs) were isolated from the testes of 6‐day‐old Sprague–Dawley (SD) rats via a two‐step enzymatic digestion and purification method as previously described [21]. The purified and enriched SSCs were maintained at 37°C with 5% CO_2_. Upon reaching 85% confluence, the SSCs were subcultured onto coverslips or 6‐well plates at a density of 1 × 10^5^ cells per square centimeter for further experiments.

### 2.4 Activation of OECs and Collection of Conditioned Medium (CM)

Primary cultures of OECs were prepared from the olfactory bulbs of 2.5‐month‐old rats and subsequently purified according to previously established methods [22]. Upon reaching the optimal density, the purified cells were subjected to digestion with 0.25% EDTA–trypsin, suspended, and equally allocated into two distinct groups. These cells were then reseeded onto either 6‐well plates for experimental assays or coverslips for immunofluorescence analysis. Typically, the same batch of OECs was evenly distributed, and one group was randomly selected as the activation group. One group was designated activated OECs (aOECs) and was cultured in medium supplemented with 1 μM conditioned medium (CCM) dissolved in DMSO, as previously described by Hao et al. [23]. The other group, designated nonaOECs, was maintained in culture medium containing an equivalent volume of DMSO as a control (solvent for CCM and LPS). To assess the potential of OECs to promote the differentiation of SSCs into spinal cord neurons, conditioned media from both OECs and aOECs were collected throughout the culture period, with supernatants harvested daily. Following this procedure, the supernatants obtained from OECs and aOECs were combined, centrifuged, and filtered through sterile membranes with a pore size of 0.45 μm to eliminate cellular debris. The conditioned media were stored at −80°C for future use.

### 2.5 Isolation and Culture of Microglia

Neonatal SD rats (clean grade) sourced from the Experimental Animal Center of Xi'an Jiaotong University within one day of birth were used for this study. For microglial culture, neonatal rats were disinfected with 75% ethanol and subsequently decapitated following anesthesia. The brains were then rapidly extracted and immersed in cold D‐Hanks solution. The meninges and blood vessels were carefully excised, and the brain tissue was finely minced prior to being transferred into a 0.25% trypsin solution for digestion at 37°C for 15 min. After the digestion was halted by the addition of DMEM containing 10% fetal bovine serum (FBS), the tissue was gently pipetted to make a cell suspension and centrifuged at 4°C for 5 min at 1000 rpm. The resulting cells were resuspended to make a single‐cell suspension and seeded at a density of 1 × 10^6^ cells/ml into 25 cm^2^ culture flasks. The cells were maintained at 37°C in a 5% CO_2_ incubator. When the cells reached over 95% confluence, the culture flasks were placed on a shaker and agitated at 37°C for 2 h at 200 rpm to obtain microglia. The detached cells were harvested, resuspended in DMEM containing 10% FBS, and transferred to new culture flasks for further culture. The cells were subcultured for further experiments when they reached 80% confluence.

### 2.6 Optimal Induction of the Transdifferentiation of SSCs Into Spinal Cord Neurons

For the induction experiments, primary spermatogonial stem cells (SSCs) were cultured at a density of 1 × 10^5^ cells/cm^2^ in 6‐well plates or on cover slips. The growth medium was replaced with DMEM/F12 supplemented with 1% FBS, which comprised either OEC‐conditioned medium (OECCM) or activated OEC‐conditioned medium (aOECCM), in conjunction with defined factors such as retinoic acid (RA), 3‐isobutyl‐1‐methylxanthine (IBMX), β‐mercaptoethanol (β‐ME), basic fibroblast growth factor (bFGF), epidermal growth factor (EGF), and leukemia inhibitory factor (LIF), in various combinations. The induction conditions were as follows: (1) D + T group (DMEM/F12 group): DMEM/F12 with 1% FBS supplemented with RA (1 × 10^7^ mol/L), IBMX (5 × 10^4^ mol/L), β‐ME (5.5 × 10^5^ mol/L), bFGF (10 ng/mL), EGF (20 ng/mL), and LIF (10 ng/mL); (2) O + T group (OEC‐CM group): OEC‐conditioned medium (OEC‐CM), DMEM/F12 supplemented with RA (1 × 10^7^ mol/L), IBMX (5 × 10^4^ mol/L), β‐ME (5.5 × 10^5^ mol/L), bFGF (10 ng/mL), EGF (20 ng/mL), and LIF (10 ng/mL); (3) A + T group (aOEC‐CM group): activated OEC‐conditioned medium (aOEC‐CM), DMEM/F12 supplemented with RA (1 × 10^7^ mol/L), IBMX (5 × 10^4^ mol/L), β‐ME (5.5 × 10^5^ ng/mL), bFGF (10 ng/mL), EGF (20 ng/mL), and LIF (7) Treatment 1 + coculture group: LPS at 0.5 μg/mL; (8) treatment 2 + coculture group: LPS = 1.0 μg/mL; (9) treatment 3 + coculture group: LPS = 2.0 μg/mL. The cells were maintained for 7, 14, or 21 days for further examination.

### 2.7 Immunofluorescence Staining

The cells cultured on coverslips or in culture dishes from all the groups were fixed with 4% paraformaldehyde for 30 min, followed by permeabilization with 0.01% Triton X‐100 (Sigma) for 10 min. Subsequently, the cells were incubated in 5% BSA solution for 1 h and incubated with primary antibodies against Tuj‐1 (1:300), Map2 (1:200), GPR125 (1:400), and UCHL1 (1:200) for 1 h, followed by overnight incubation at 4°C with appropriate dilutions. All subsequent procedures and image capture were performed according to previously reported methods [24].

### 2.8 RT–PCR and qPCR Analysis

Total RNA extraction, reverse transcription, and PCR were conducted in accordance with the manufacturer's instructions via standard procedures. GAPDH served as an internal control, and the expression levels of target mRNAs were normalized to those of GAPDH. The target genes selected for analysis included Tuj1, Map2, NeuN, UCHL1, Vglut2, GABA, TH, Chat, Ache, A1dh1a1, TPH2, Calretinin, DAZL, and PLZF. The primer sequences and the corresponding amplification products for RT–PCR are shown in Supporting Information S1. Data analysis was conducted according to previously described methods [25].

### 2.9 Western Blots

Protein extracts were obtained from cells and tissues subjected to different treatments and subsequently analyzed according to previously described protocols. The primary antibodies used in this study included those against MAP2 (1:1000), Tuj‐1 (1:1000), NeuN (1:1000), GPR125 (1:1000), UCHL1 (1:1000), JAK2 (1:1000), STAT3 (1:1000), P‐JAK2 (1:1000), and P‐STAT3 (1:2000). The dilutions of all primary antibodies were prepared in accordance with the manufacturer's instructions or with minor modifications where necessary. GAPDH served as an internal control. After thorough washing in PBS, the immunoblots were visualized via enhanced chemiluminescence, and imaging was performed with a gel imaging system camera.

### 2.10 LPS‐Induced Activation of Microglia and Coculture of Activated OECs and SSCs In Vitro

Activated OECs and SSCs were mixed at a ratio of 1:5, yielding a total cell density of 1 × 10^6^ cells/mL. This cell mixture was subsequently seeded into the lower chamber of a Transwell system, corresponding to the bottom of the culture plate, and supplemented with an appropriate differentiation induction medium. Concurrently, primary microglia were cultured until they reached 80%–90% confluence, at which point they were seeded into the upper chamber of the Transwell system or the insert chamber at a density of 1 × 10^5^ cells/mL. To activate microglia, LPS was added to the microglial culture medium at various concentrations (0.5 μg/mL, 1 μg/mL, and 2 μg/mL). The cultures were maintained at 37°C in a 5% CO_2_ incubator for 24 h. The Transwell chambers containing microglia exposed to different concentrations of LPS were subsequently transferred to a 6‐well plate containing activated OECs and SSCs, which had been subjected to induction for various durations. The coculture system was then incubated at 37°C in a 5% CO_2_ incubator for 72 h to observe the interactions between the cells.

### 2.11 Electrophysiology

Three weeks after the induction of SSC differentiation, the differentiated cells were transferred to artificial cerebrospinal fluid (ACSF) and subjected to ventilation with a gas mixture of 95% O_2_ and 5% CO_2_ to evaluate their ability to generate action potentials. The preparation of ACSF followed previously established protocols. Whole‐cell patch‐clamp experiments were performed via an inverted microscope (Zeiss, Jena, Germany) maintained at a temperature range of 20°C to 22°C. For current–clamp recordings, a 10 pA current pulse was administered to assess the ability of the cells to generate action potentials. Cells exhibiting a leak current of less than 100 pA were selected for further analysis. Whole‐cell currents were filtered via a 2.9 kHz low‐pass filter and digitized at a 10 kHz sampling rate via an EPC‐10 amplifier (HEKA, Lambrecht, Germany), and the data were analyzed via PATCHMASTER software (HEKA). Tetrodotoxin (TTX) at a concentration of 0.2 mM (Sigma–Aldrich) was diluted in the bath solution and applied via an SF‐77B fast‐step perfusion system by gravity, as previously described. In voltage‐clamp mode, a series of voltage steps ranging from −50 to +50 mV in 10‐mV increments were employed to evoke and record whole‐cell sodium and potassium currents. Action potentials (APs) were recorded in voltage‐step mode.

### 2.12 Calcium Imaging

To evaluate the functional properties of neurons derived from SSCs, we investigated calcium ion influx in individual cells via previously described methods[?]. In brief, the stem cells were subjected to induction under specified conditions for 3 weeks. Following this period, the differentiated cells were transferred to neurobasal medium supplemented with 1% B27 and 2 mM of the calcium‐sensitive fluorescent indicator Fluo 4‐AM (Sigma–Aldrich) and incubated at 37°C for 30 min. Subsequently, the cells were thoroughly washed twice with extracellular medium (EM; comprising 140 mM NaCl, 2 mM CaCl2, 5 mM KCl, 10 mM HEPES, and 10 mM glucose, with the pH adjusted to 7.2–7.4) within a perfusion chamber. Calcium imaging was conducted via EM using a Zeiss LSM 710 confocal microscope. To specifically assess Ca^2+^ influx, a Ca^2+^ channel activator (10 mM BayK; Stemgent) or blocker (5 mM nifedipine; Sigma–Aldrich) was introduced to the culture to monitor alterations in Ca^2+^ influx. This procedure was executed in accordance with our previously published protocol. To further characterize the physiological properties of the induced neurons, 100 mM KCl was administered to the cells following nifedipine treatment, and subsequent Ca^2+^ influx images were obtained.

### 2.13 Animals and Experimental Groupings

A total of 48 female SD rats weighing 200 ± 30 g were obtained from the Experimental Animal Center at Xi'an Jiaotong University. The rats were housed in an environment with a stable temperature of 25°C ± 2°C and a humidity level of 50% ± 10% under a 12‐h light/dark cycle with continuous air exchange. The animals were randomly divided into four groups: (1) the control group, in which rats underwent only laminectomy and compressive SCI; (2) the SCI + 24 h group, in which activated (aOECs) and differentiated neuronal cell concentrated suspensions were transplanted 24 h post‐SCI; (3) the SCI + 72 h group, in which OECs and differentiated neuronal cell concentrated suspensions were transplanted 72 h post‐SCI; and (4) the SCI + 7 days group, in which aOECs and differentiated neuronal cell concentrated suspensions were transplanted 7 days post‐SCI. Twelve experimental animals were utilized in each group to conduct behavioral and morphological assessments following the aforementioned surgical procedures.

### 2.14 Animal SCI Model and Transplantation of Cells

Twenty minutes prior to surgery, each group of rats was anesthetized via isoflurane administered through a veterinary anesthesia machine (RWD, Shenzhen, Guangdong Province, China). Following isoflurane administration, the rats were further given an intraperitoneal injection of 2% pentobarbital sodium at a dosage of 30 mg/kg to induce further anesthesia. Simultaneously, the rats were positioned in a prone orientation. To prevent ocular desiccation during the procedure, petroleum jelly was applied to the eyes of the patients. After the animals' fur was removed, the surgical site was disinfected with medical iodine. The surgical method for inducing a compressive SCI model was performed as previously described [26]. A longitudinal incision was made at the T7–11 region via sterile surgical scissors. The paravertebral muscles were dissected beneath the periosteum, and a laminectomy was performed to expose the spinal cord at T9 or T10. In the exposed spinal cord region, a calibrated compression method was employed with microsurgical forceps (Yunkang, Jiangsu Province, China) to induce compressive injury, which was maintained for 20 s before the forceps were carefully removed [27]. During compression, the tip spacing of the forceps was set at 1 mm.

The rats were positioned using a stereotaxic frame (RWD) for the transplantation of SSCs. Primary culture of olfactory sheath cells was performed using GFP‐labeled transgenic SD rat strains. After passaging and purification, the olfactory sheath cells were activated with cell culture medium (CCM). The activated olfactory sheath cell suspension and differentiated spinal neuron suspension were aspirated via a microinjector, and the activated olfactory sheath cells were mixed with the spinal neurons at a ratio of 1:5, resulting in a final cell suspension volume of 10 μL. The cell suspension was gently mixed in a sterile environment to ensure the absence of bubbles. A 10 μL cell suspension containing 2 × 10^5^ cells or saline (SCI group) was injected into the core of the injured spinal cord via a 10 μL silanized Hamilton syringe equipped with a beveled glass capillary tip (inner diameter 80 × 90 μm). The injection was conducted at a consistent and slow rate of 0.2 μL/min, with the glass capillary held in position for an additional 5 min postinjection to prevent leakage prior to withdrawal. The entire cell transplantation process took approximately 30 min [28]. The incision was subsequently rinsed with sterile saline, and the tissue layers were subsequently sutured. Postoperatively, glucose was added to the water supplied to the rats to provide energy, and cefuroxime was administered to prevent infection. Manual bladder expression was performed daily, and the feeding amount was gradually adjusted on the basis of the animal's behavior and recovery status.

### 2.15 Behavioral Assessment

Behavioral assessments were conducted on the experimental rats both prior to surgery and at 1, 2, and 3 weeks postsurgery, utilizing the Basso‐Beattie‐Bresnahan (BBB) locomotor scoring scale to quantify motor function. This scale is highly sensitive to various parameters, including joint mobility of the hind limbs, hind limb movement capability, trunk posture and stability, coordination, gait, paw placement, and tail posture. The detailed scoring criteria can be found in the relevant literature [29, 30, 31]. An improvement in the BBB score is positively correlated with the degree of motor function recovery, which can be subdivided into early, middle, and late stages. In the experiment, each rat was individually placed on a 90 cm plastic platform, where two evaluators observed and scored them, with a scoring duration of 4 min per rat, and each hind limb was assessed independently. The final score for each limb was determined as the lowest score assigned by the two observers. The scoring range for the rats ranged from 0 to 21, where a score of 0 indicated no movement in the hind limbs, whereas a score of 21 indicated complete recovery of hind limb movement, characterized by a wide range of joint mobility, including full extension of the hind limbs, movement of the knee and hip joints, coordinated paw pedaling movements, consistent with clearance, absence of rotation during pedaling, and sustained upward movement of the tail [29, 30, 31]. All behavioral assessments were conducted by two researchers who were blinded to the severity of the injury. The rats were grouped on the basis of the extent of their injuries, and the average scores for each group were calculated. The Basso, Beattie, and Bresnahan (BBB) scores were subsequently analyzed in relation to the duration of the injury.

### 2.16 Evoked Potentials

Prior to surgery and throughout the first 3 weeks following transplantation, motor evoked potentials (MEPs) in the rats were systematically recorded, and motor nerve conduction function was evaluated via the Nicolet EDX electromyography evoked potential system. Under general anesthesia induced with 2% pentobarbital sodium at a dosage of 30 mg/kg, the sensorimotor cortex (SMC) and sciatic nerve were surgically exposed, and stimulation and recording electrodes were appropriately connected. MEPs were elicited via electrical stimulation of the SMC, which was positioned 2 mm lateral to the midline and 2 mm posterior to the pons, using a single pulse of 50 ms duration. The voltage was calibrated according to experimental requirements to achieve the maximum MEP amplitude, typically ranging from 6 to 10 V under normal physiological conditions. The amplitude and latency of the MEPs were subsequently measured with precision.

In this study, all experimental rats initially underwent somatosensory evoked potential (SEP) testing of the left forelimb, which was identified as the affected side, to establish baseline data. Subsequent SEP monitoring was conducted during the first, second, and third weeks following stem cell transplantation. The monitoring procedure involved anesthetizing the animals to facilitate their transition into the experimental state. During this process, the rats' heads were immobilized, and the necessary preparations were performed. Recording electrodes were strategically positioned along the interparietal bone and periosteum at the junction of the parietal bones on both sides of the skull, with the left recording electrode specifically placed over the right hemisphere of the brain. A reference electrode was affixed to the nose, a ground electrode was affixed to the ear, and a stimulation electrode was inserted into the toes of the left forelimb. The interelectrode distance was maintained at approximately 1 cm. These electrodes were then sequentially connected to the amplifier and stimulation interfaces.

### 2.17 Cell Counts

Under a fluorescence microscope, 15 randomly selected fields of view on each cover slip were examined at approximately 20× magnification. Each observed field encompassed an area of approximately 0.45 square millimeters. Upon terminating the culture at predetermined time points, the neurons were counted according to established methodologies. For each experimental group, four cover slips were used for counting analysis. Notably, to ensure objectivity between the experimental and control groups, counting was performed by an individual who was blinded to the experimental details, adhering to a standardized counting protocol, with each sample being counted twice.

### 2.18 Statistical Analysis

All values obtained from the analyzes are expressed as the means ± standard errors of the means (SEM). Statistical analysis was conducted via SPSS 10.0 statistical software, with significant differences between groups under different conditions assessed via analysis of variance (ANOVA), where *p* < 0.05 was considered statistically significant.

## 3 Results

### 3.1 Identification of SSCs and the Transdifferentiation Induction System

**FIGURE 1:**
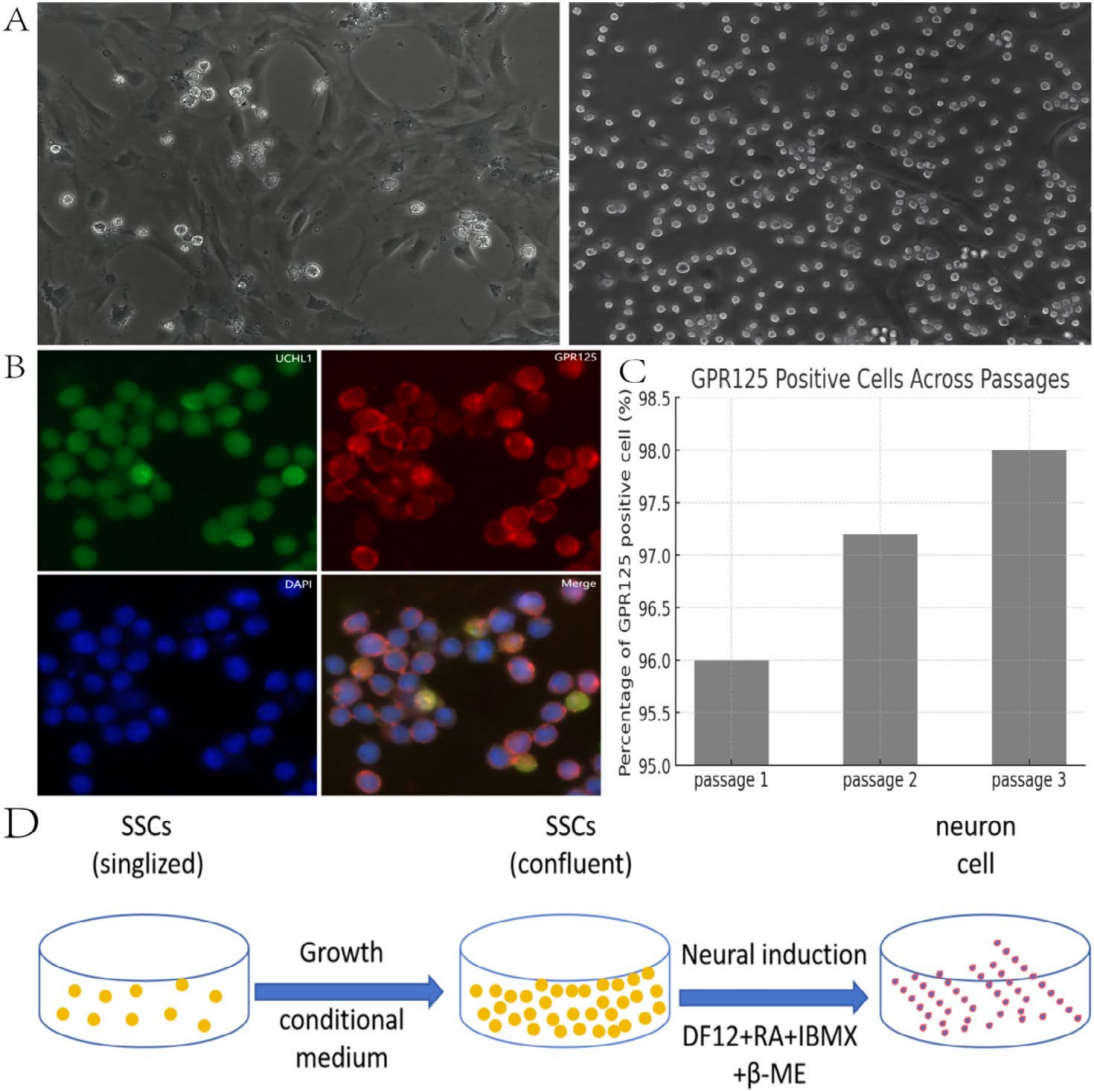
Schematic of the characterization and differentiation of spermatogonial stem cells (SSCs). (A) The upper left section of the panel represents the primary SSCs, whereas the upper right of the panel depicts subcultured SSCs after purification. (B) Immunofluorescence microscopy images of primary SSCs labeled with the indicated antibodies. (C) Quantitative assessment of the number of GPR125‐positive cells following purification and subculturing from passages 1 to 3. Notably, the purity of the SSCs was calculated as the ratio of the number of GPR125‐positive cells to the total number of DAPI‐stained nuclei. (D) Schematic diagram illustrating the differentiation system used to induce the differentiation of spinal cord neurons from SSCs.

After the SSCs were cultured in flasks coated with PLL and gelatin for more than three days, phase‐contrast microscopy revealed that the primary SSCs formed grape‐like clusters on the sheet‐like cells. On the basis of their morphological features, these underlying cells were predominantly somatic cells and Sertoli cells (Figure 1A, left panel). By the seventh day, the primary cells exhibited a pronounced colony‐like morphology, with an observable expansion in both volume and a gradual increase, and the number of cell clusters increased and expanded gradually. Upon detachment and purification, these cells appeared round and uniform, displaying the typical morphological characteristics of SSCs (Figure 1A, right panel). Double immunostaining demonstrated that the freshly detached cells from the underlying layer were positive for GFR125 and UCHL1 (Figure 1B). Furthermore, quantitative analyzes indicated that GFR125‐positive cells maintained high purity, exceeding 96%, even after undergoing at least three subcultures (Figure 1C).

For the induction experiments, primary SSCs were cultured at a density of 1 × 10^5^ cells/cm^2^ in 6‐well plates or coverslips. The experimental outline for the differentiation of SSCs is depicted in Figure 1D. The growth medium was subsequently replaced with induction‐conditioned medium. After 21 days of induction culture, the cells were further examined.

### 3.2 Identification of the Transdifferentiation of SSCs Into Spinal Cord Neurons via Optimal Culture Media

**FIGURE 2:**
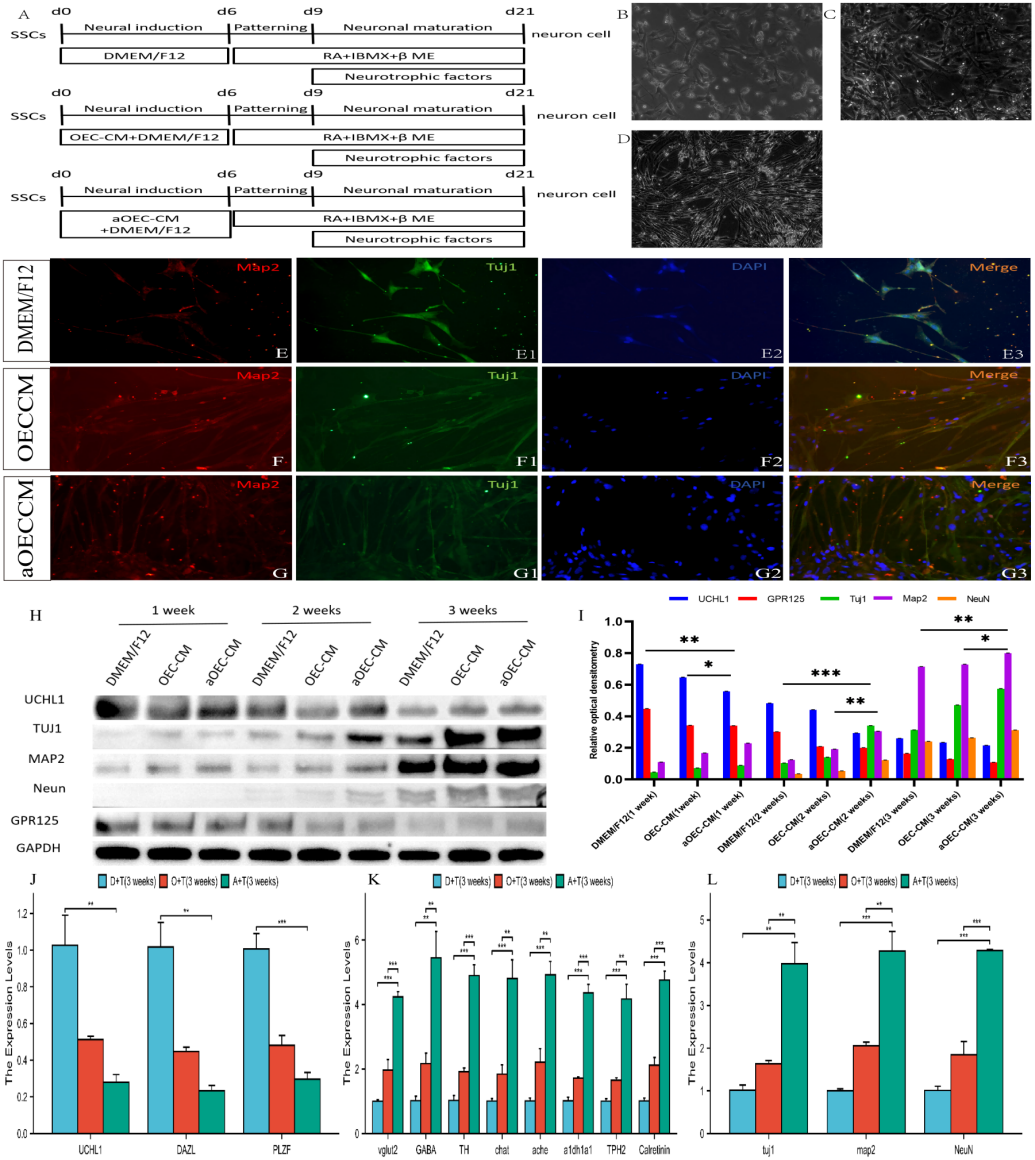
Comparative analysis of the protocol for the differentiation of SSCs into spinal neurons and biochemical phenotypic profiling. (A) Schematic diagram illustrating the strategies employed in the three induction protocols. (B–D) Morphological features of primary SSCs under distinct induction conditions. (B) DMEM/F12; (C) OEC‐CM induction; (D) AOEC‐CM induction. (E–G) Immunofluorescent characteristics of SSCs under three distinct induction conditions over a period of 3 weeks. Representative images showing the expression of the neuronal cell‐specific markers MAP2 and Tuj‐1 in differentiated cells. (H) Western blot analysis of the temporal variations in stage‐specific markers during the neuronal differentiation of SSCs under defined conditions. (I) Quantification of the expression levels of stage‐specific markers normalized to those of GAPDH. *n* = 10. (J–L) Quantitative real‐time PCR analysis of selected genes involved in cellular functions and stem cell characteristics in SSCs subjected to various induction protocols at 3 weeks postdifferentiation. *p < 0.05, **p < 0.01, ***p < 0.005.

For the differentiation of spinal neurons from SSCs, the cells were cultured on PLL/gelatin‐coated plates and treated with RA, IBMX, β‐ME, and neurotrophic factors (GDNF, EGF, bFGF, LIF), as schematized in Figure 2A. Over 3 weeks of induction, the neural morphology of the SSCs progressively acquired, characterized by neurite outgrowth (Figure 2B–D). Immunofluorescence analysis revealed that the activated OEC‐CM group exhibited enhanced neuronal differentiation, with higher expression levels of Tuj‐1 and MAP2 than the DMEM/F12 and OEC‐CM groups did (Figure 2E–G, Supporting Information S1). Western blot analysis revealed a progressive reduction in the expression of SSC markers (UCHL1/GPR125) and a marked increase in the expression of neuronal markers (Tuj‐1/Map2/NeuN) in the aOEC‐CM group compared with the control group (*p* < 0.05, Figure 2H,I). Complementary qRT–PCR analysis confirmed these findings, revealing significantly increased expression of both panneuronal markers and subtype‐specific markers (GABA/ChAT/AchE) in aOEC‐CM‐treated cells (*p* < 0.05, Figure 2J–L, Supporting Information S2), validating the optimal neural differentiation capacity of this protocol.

### 3.3 Identification of the Transdifferentiation of SSCs Into Spinal Neurons Induced by Three Retinoic Acid (RA) Time‐Dependent Systems

**FIGURE 3:**
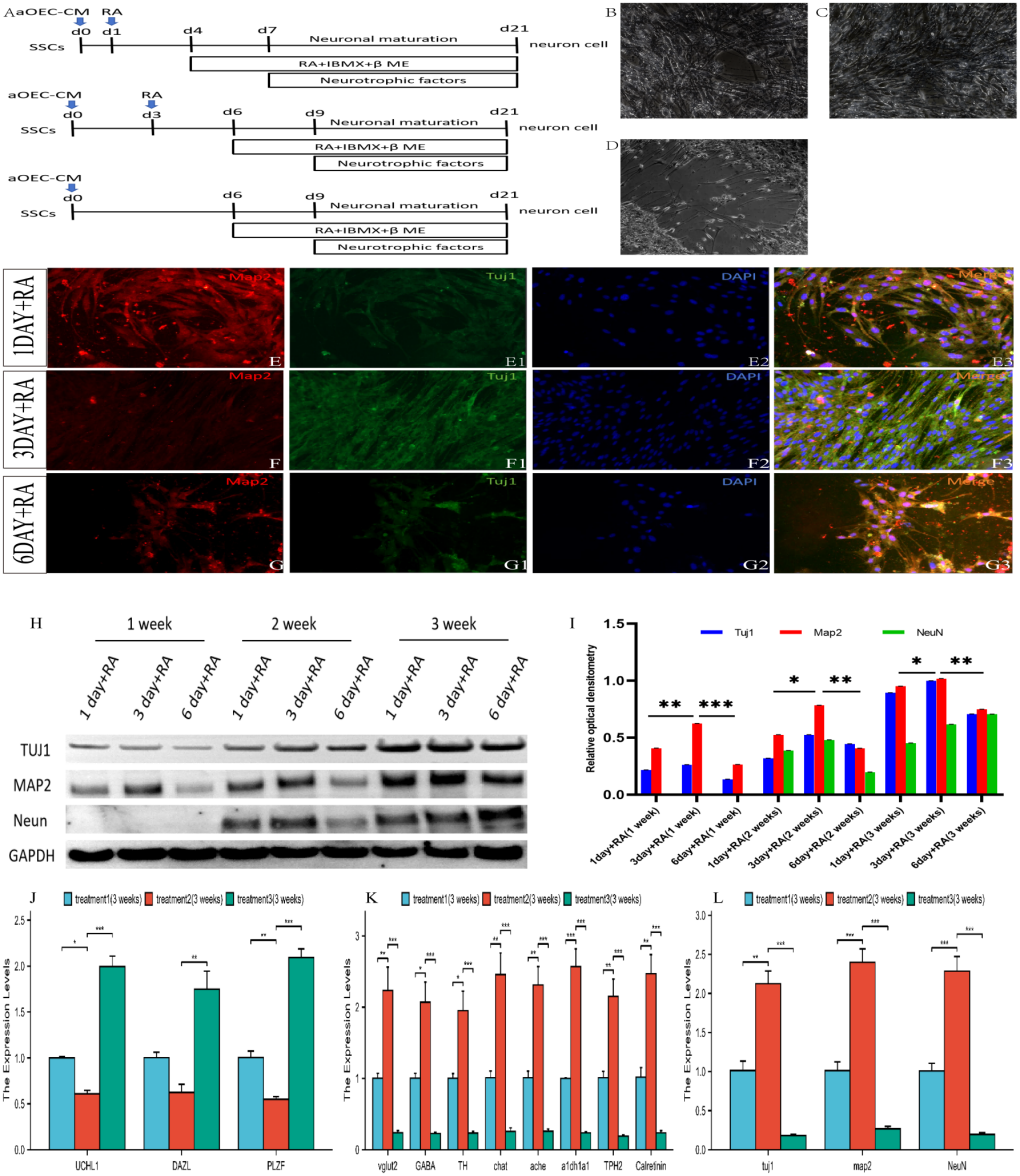
Identification of the transdifferentiation of SSCs into spinal cord neurons under various temporal induction protocols and analysis of their phenotypic characteristics. (A) Schematic of three strategies for differentiating SSCs into spinal cord neurons. Notably, all protocols incorporate RA and aOEC‐CM during the neural induction phase on days 1, 3, and 6. (B–D) Morphology of SSC‐derived spinal cord neurons under distinct RA induction windows (1/3/6 days). Phase contrast images: (B) 1‐day RA induction scheme, (C) 3‐day scheme, (D) 6‐day scheme. (E–G3) Immunofluorescence identification images for the three induction schemes. Immunofluorescence microscopic photographs used to identify cells and their specific markers; the indicated antibodies are shown in the figure. (H, I) Western blot analysis of neuronal markers (Tuj1/Map2/Neun) across three induction schemes: (H) temporal expression profiles (weeks 1–3), (I) quantitative protein levels. (J–L) qRT–PCR quantification of lineage‐specific genes (pluripotency/neuronal/glial markers) in NSCs and SSCs after 3 weeks of differentiation across induction protocols. *p < 0.05, **p < 0.01, ***p < 0.005.

SSCs cultured on PLL/gelatin‐coated plates were subjected to RA administration at three time points (days 1, 3, and 6) to evaluate their neuronal marker expression profiles (Figure 3A). Morphological and immunofluorescence analyzes revealed that, compared with treatment on days 1 and 6, day 3 of RA administration resulted in optimal spinal differentiation into spinal neurons (Figure 3B–D), as evidenced by significantly increased expression of Tuj1, Map2, and NeuN. This finding highlights the critical role of temporal RA regulation in enhancing neuronal differentiation efficiency (Figure 3F,G). Delayed RA administration on day 6 was found to impair motor neuron differentiation. In contrast, initiating RA treatment on day 3 in combination with aOEC‐CM treatment demonstrated optimal efficacy. This protocol yielded a 2.3‐fold increase in Tuj1/Map2‐positive cells compared with those in the treatment groups on days 1 and 6, accompanied by corresponding 1.8‐ to 2.5‐fold increases in neuronal marker protein levels, as determined by Western blot analysis, and in mRNA levels, as assessed by qRT–PCR (Figure 3H,I, Supporting Information S3). These results confirm that precise temporal regulation by RA maximizes the conversion efficiency of SSCs to neurons.

### 3.4 Neurons Derived From SSCs Exhibit Characteristic Electrophysiological Properties

**FIGURE 4:**
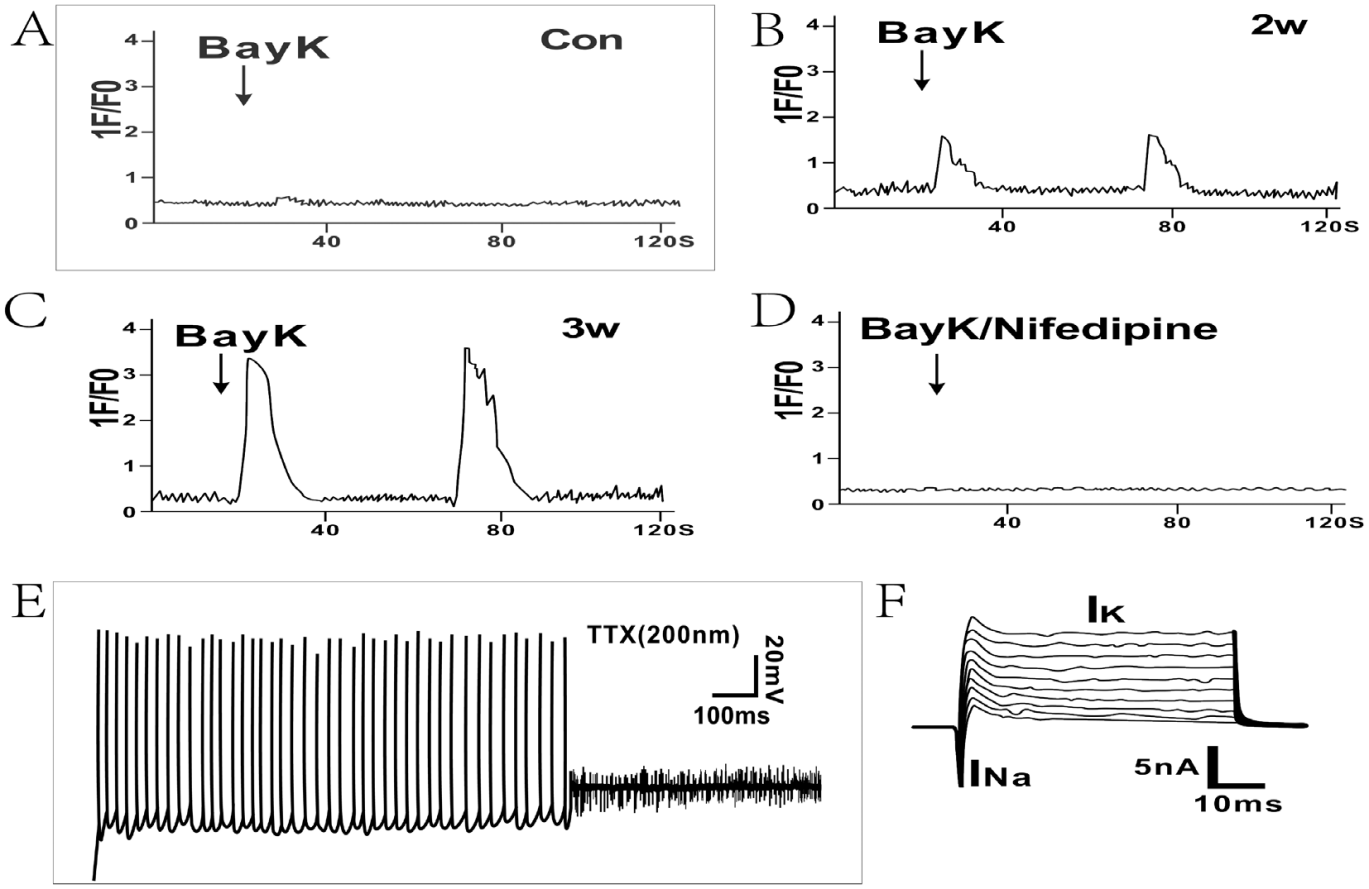
Functional profiling of neurons derived from SSCs. (A–D) Functional characteristics of the L‐type calcium channel. Changes in the calcium response in differentiated neurons ((A) SSCs; (B, C, and D) differentiated neurons derived from 2‐week‐ and 3‐week‐induced SSCs, respectively) with or without nifedipine after treatment with BayK. 1F/F0 indicates the fluorescence intensity ratio between the baseline (0 s) and stimulus phases. (E) Current–clamp recordings of action potentials evoked in SSC‐derived neurons following 3 weeks of induction. The action potentials were suppressed by the application of TTX. (F) Voltage–clamp recording of SSC‐derived neurons. Depolarizing sodium and potassium currents are evoked by the membrane potential at various levels.

To further evaluate the functionality of neurons derived from SSCs within the optimized high‐differentiation efficiency induction system, we conducted calcium imaging experiments. After a differentiation period of 2–3 weeks, these neurons displayed typical characteristic neuronal calcium influx. As shown in Figure 4A–D, exposure to the calcium channel agonist BayK resulted in a significant increase in intracellular calcium influx in the induced neurons. Specifically, after 2 weeks of induction, the fluorescence intensity increased approximately sixfold, and after 3 weeks, it increased approximately eightfold. Notably, even after cessation of BayK treatment, the converted neurons maintained elevated fluorescence intensity, which was subsequently attenuated by the calcium channel blocker nifedipine. These results suggest that differentiated neurons exhibit the typical physiological activity characteristics of neurons. Furthermore, 3 weeks postdifferentiation, we recorded the electrophysiological properties of these neurons via whole‐cell patch‐clamp techniques. As shown in Figure 4E,F, during current‐clamp experiments, the application of the sodium channel blocker tetrodotoxin (TTX) did not completely inhibit the generation of spontaneous action potentials (APs) in some cells, indicating that these differentiated cells exhibit a certain degree of maturity in terms of neuronal functionality. Furthermore, in whole‐cell patch‐clamp mode, the differentiated cells presented substantial sodium and potassium ion currents (Figure 4F), confirming their functional neuronal properties.

### 3.5 Activated OECs Promote the Differentiation of SSCs Into Spinal Cord Neurons Under Conditions of LPS‐Induced Microglial Inflammation

**FIGURE 5:**
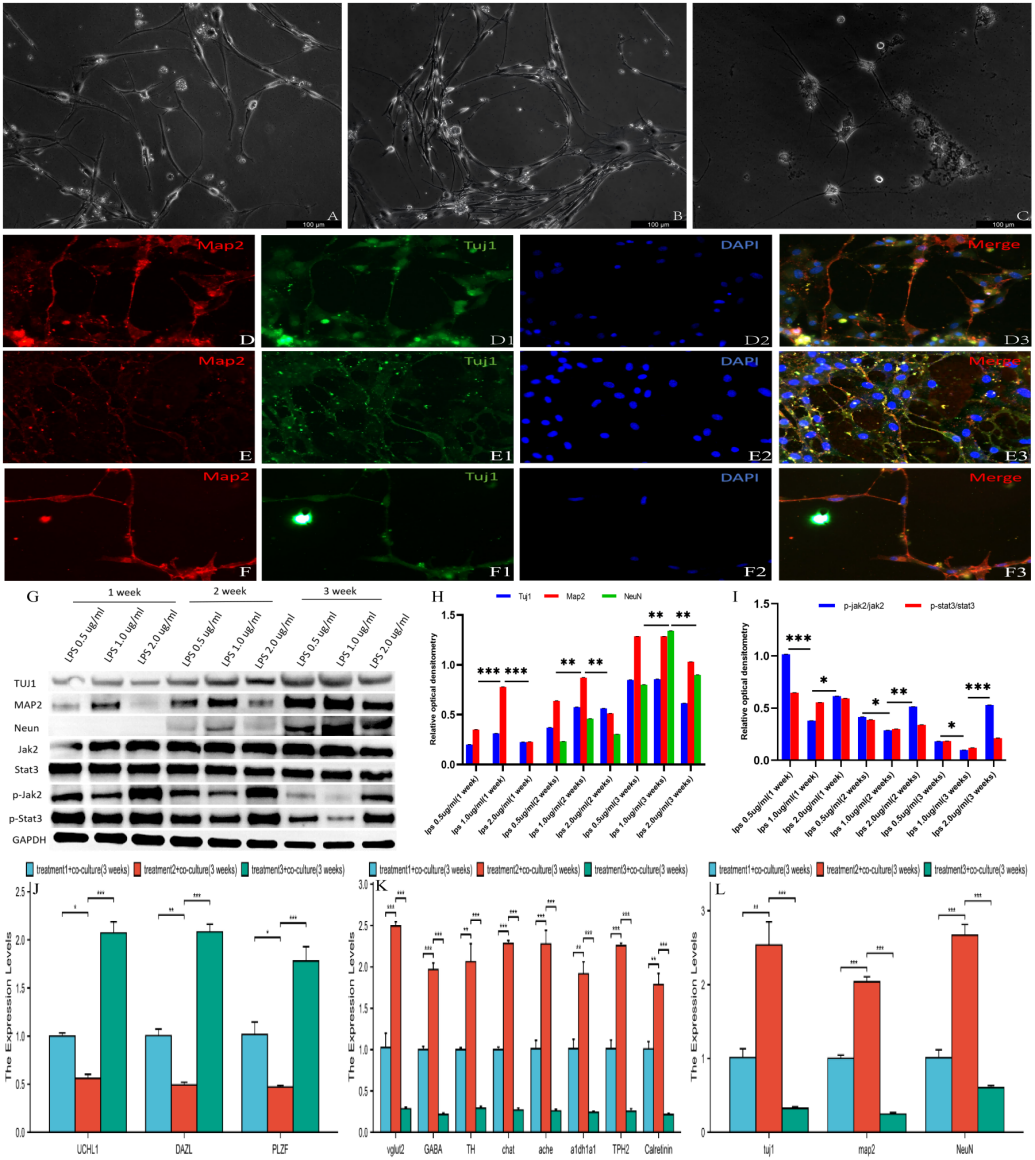
Immunofluorescence analysis and phenotypic characteristics of transdifferentiated spinal neurons derived from SSCs under inflammatory conditions in the presence of aOECs. (A–C) Morphological analysis of the effects of different doses of LPS on differentiated spinal neurons. (A) 0.5 μg/mL, (B) 1.0 μg/mL, and (C) 2.0 μg/mL. (D–F) Immunofluorescence analysis of LPS‐induced differentiation in SSCs revealed dose‐dependent changes in the expression of neuronal markers. Specifically, the groups were as follows: (D–D3) 0.5 μg/mL; (E–E3) 1.0 μg/mL; and (F–F3) 2.0 μg/mL. (G) Temporal immunoblot analysis of the expression profiles of neural markers, including Tuj1, Map2, and Neun, as well as components of the JAK–STAT signaling pathway, specifically JAK2, STAT3, p‐JAK2, and p‐STAT3, during 3 weeks of differentiation via defined induction protocols. (H) Quantitative analysis of the expression levels of the neuronal maturation markers Tuj1, Map2, and NeuN in chemically induced cellular models. (I) Determination of the JAK–STAT activation index through ratiometric analysis of phosphorylated‐to‐total protein levels in neural differentiation models. (J–L) qRT–PCR analyzes of the transcripts of trilineage neural markers in SSCs exposed to the TNFα/IL‐1β inflammatory mixture. *p < 0.05, **p < 0.01, ***p < 0.005.

In microglial inflammation models induced by lipopolysaccharide (LPS), optimal neuronal differentiation was achieved at an LPS concentration of 1.0 μg/mL, as demonstrated by a 2.3‐fold increase in Tuj1/Map2‐positive cells compared with those in the other LPS concentration groups. Notably, this differentiation efficiency exhibited a dose‐dependent relationship with LPS, as evidenced by differentiation rates of 68.7% ± 5.2% in the 1.0 μg/mL LPS group, compared with 29.4% ± 3.8% in the 0.5 μg/mL group and 41.2% ± 4.1% in the 2.0 μg/mL group, under conditions of controlled neuroinflammation. These results validated the ability of the activated OEC induction system to moderate inflammatory microenvironments during spinal neuron differentiation (Figure 5A–F). Compared with the other groups, the group treated with 1.0 μg/mL LPS demonstrated sustained suppression of JAK2/STAT3 phosphorylation, with a 42% reduction in JAK2/STAT3 phosphorylation, along with a 3.1‐fold increase in the expression of the neuronal markers Tuj‐1, Map2, and NeuN throughout the induction period (*p* < 0.05, Figure 5G–I). qRT–PCR analysis revealed an increase in neural and glial marker transcription (GABA: 4.8 ± 0.3‐fold; AChE: 3.9 ± 0.2‐fold) and a concurrent 60%–75% reduction in the expression of stemness markers (UCHL1, DAZL, and PLZF), confirming that 1.0 μg/mL LPS optimally balances inflammatory signaling and neurogenic potential within the OEC coculture induction system (Figure 5J–L, Supporting Information S4).

### 3.6 Influence of the Activated OEC Induction System on the Differentiation of SSCs Into Spinal Cord Neurons via the JAK2/STAT3 Signaling Pathway

**FIGURE 6:**
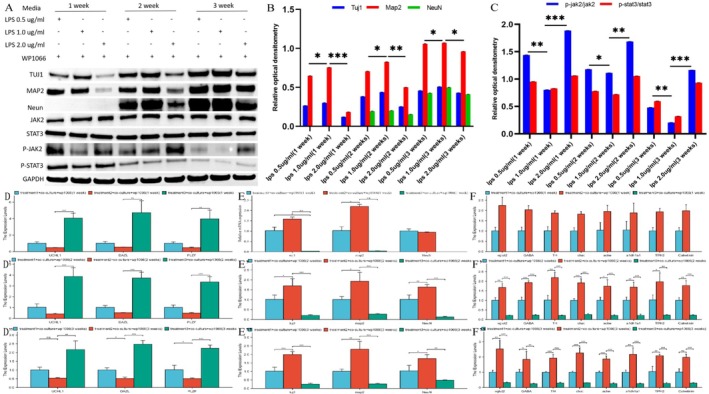
The inhibition of JAK/STAT signaling mediated by WP1066 influences the transdifferentiation of spinal cord neurons in coculture models. (A) Immunoblotting analysis revealed the temporal expression patterns of neuronal markers (TUJ1, MAP2, and NeuN) and components of the JAK/STAT signaling pathway (JAK2, STAT3, p‐JAK2, and p‐STAT3) over the course of weeks 1 to 3 of differentiation under specified conditions. (B) Quantitative analysis of neuronal differentiation markers, including TUJ1, MAP2, and NeuN, in conditionally induced neurons. (C) Analysis of JAK/STAT signaling pathway activation in conditionally induced neurons. (D–F2) JAK/STAT‐dependent transcriptional changes during neural lineage specification under WP1066‐mediated pharmacological inhibition. (D–D2) Quantitative analysis of the mRNA levels of SSC, neuronal, and glial markers at 1 week postdifferentiation. (E–E2) Quantitative analysis of the mRNA levels of SSC, neuronal, and glial markers at 2 weeks postdifferentiation. (F–F2) Quantitative analysis of the mRNA levels of SSC, neuronal, and glial markers at 3 weeks postdifferentiation. *p < 0.05, **p < 0.01, ***p < 0.005.

Inhibition of the JAK/STAT pathway in cocultures simulating spinal injury revealed a dual regulatory effect: compared with the other groups, the groups treated with 1.0 μg/mL LPS presented a 58% reduction in p‐JAK2/STAT3 levels but a 2.3‐fold increase in the expression of the neuronal markers Tuj‐1, Map2, and NeuN (*p* < 0.05) (Figure 6A–C). Treatment with WP1066 further enhanced this effect, resulting in a 3.1‐fold increase in neuronal marker expression along the side and a 72% suppression of the pathway, demonstrating that targeted inhibition of JAK/STAT promotes the neurogenic conversion of SSCs within inflammatory microenvironments. qRT–PCR analysis revealed that WP1066‐mediated JAK/STAT inhibition selectively increased neuronal transcription, with 1.0 μg/mL LPS cocultures showing a 3.2‐fold increase in Tuj1, Map2, and NeuN expression relative to other concentrations (*p* < 0.05, Figure 6D–F). These findings indicate that precise modulation of inflammation via this pathway optimizes the efficiency of SSC neurogenic reprogramming.

### 3.7 Motor Scores in Rat Models Following SSC Transplantation

**FIGURE 7:**
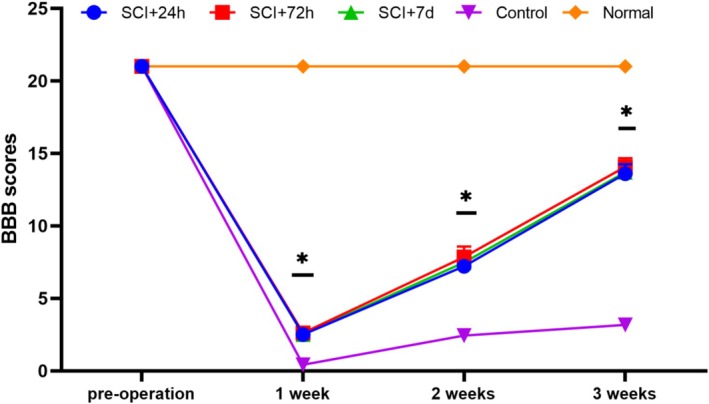
Effects of the transplantation of SSC‐derived neuronal cells on improvements in behavioral deficits in a rat model of SCI. Assessment of the temporal profile of BBB scores in experimental rat cohorts during pretreatment and posttreatment intervals. Notably, locomotor recovery in recipients reached its peak at 72 h postsurgery, exceeding the recovery observed during both the acute phase (24 h) and the subacute phase (7 days). Statistical significance is indicated as follows: *p < 0.05, **p < 0.01, ***p < 0.005.

We employed the BBB scoring system to evaluate the recovery of hind limb motor function following SCI in rats. In this scoring system, higher scores correspond to improved functional recovery. Compared with the mock group, all three experimental groups presented a significant loss of motor function on the first postoperative day, confirming successful establishment of the rat SCI model. Notably, the SCI + 72 h group demonstrated a significantly faster rate of functional recovery than the control group, SCI + 24 h group, and SCI + 7 days group did, and this difference reached statistical significance (*p* < 0.05). This result further highlights the beneficial role of stem cell transplantation during the nonacute phase in the repair of spinal cord injuries. Three weeks postsurgery, the BBB scores for each group were as follows: SCI + 24 h group (13.61 ± 0.64), SCI + 72 h group (14.08 ± 0.60), SCI + 7 days group (13.70 ± 0.65), and control group (3.18 ± 0.11). These results indicate that stem cell transplantation during the nonacute phase significantly enhances recovery outcomes in rats with spinal cord injuries, resulting in a more pronounced recovery than that in the acute phase treatment or control groups (Table 1, Figure 7). The immunofluorescence of cell therapy in spinal cord injury rat models matching the BBB scoring period is presented in Supporting Information S11.

**TABLE 1:** BBB score of the transplantation group versus the simple injury group.

Group	Preoperation	1 week	2 weeks	3 weeks
Normal	21.00 ± 0.00	21.00 ± 0.00	21.00 ± 0.00	21.00 ± 0.00
Control	21.00 ± 0.00	0.42 ± 0.08^a^	2.44 ± 0.20^a^	3.18 ± 0.11^a^
SSC + 24 h	21.00 ± 0.00	2.51 ± 0.46^a^, ^b^	7.21 ± 0.22^a^, ^b^	13.61 ± 0.64^a^, ^b^
SSC + 72 h	21.00 ± 0.00	2.62 ± 0.22^a^, ^b^	7.86 ± 0.72^a^, ^b^	14.08 ± 0.60^a^, ^b^
SSC + 7d	21.00 ± 0.00	2.51 ± 0.35^a^, ^b^	7.48 ± 0.25^a^, ^b^	13.70 ± 0.65^a^, ^b^
*p*		< 0.05	< 0.05	< 0.05

^a^ *p* < 0.05 for comparisons of the other groups with the normal group at the same time point.

^b^ *p* < 0.05 for comparisons of the experimental group with the control group at the same time point.

### 3.8 Electrophysiological Changes in Evoked Potentials of Rat Models Following Transplantation of Spinal Cord Neurons Derived From Differentiated SSCs

**FIGURE 8:**
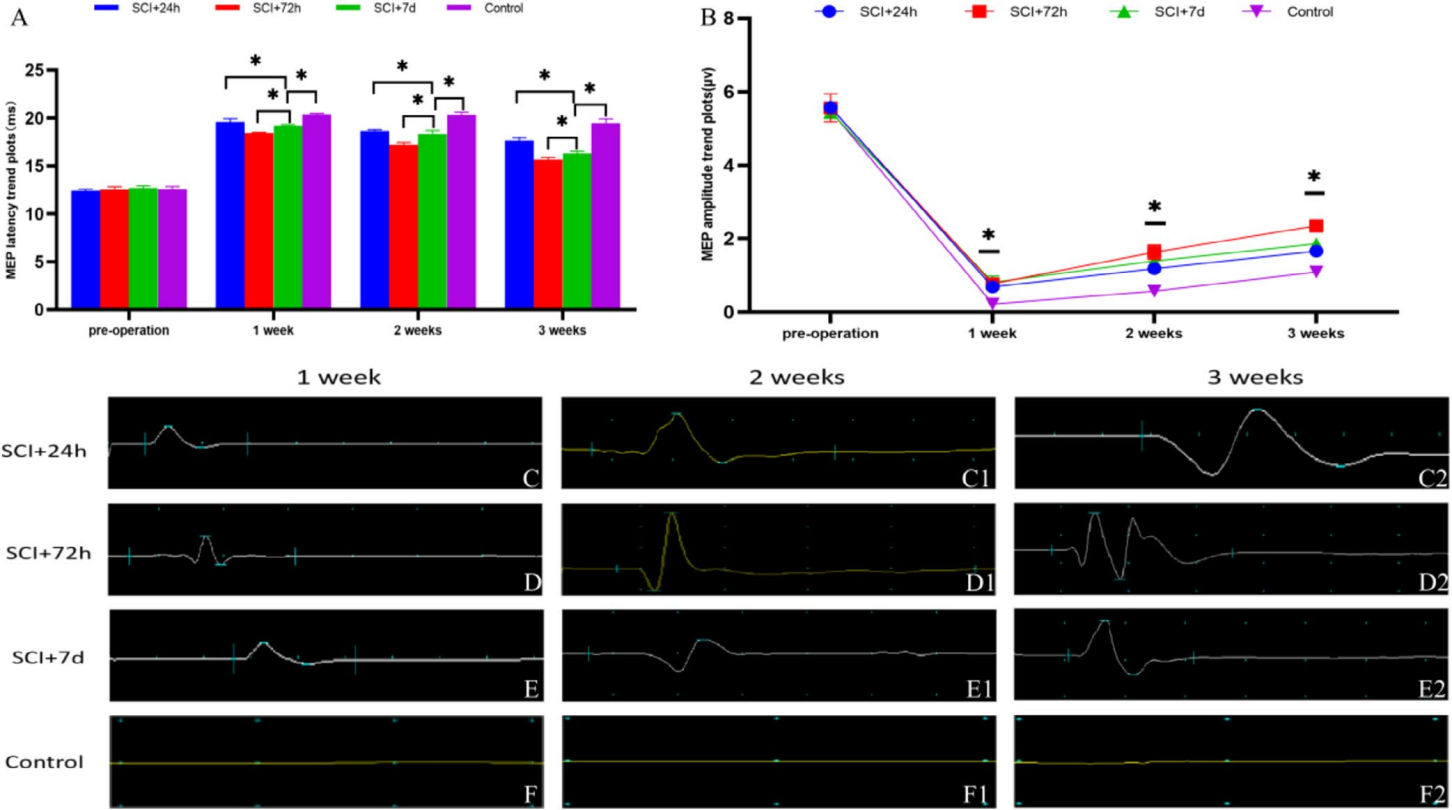
Effects of the transplantation of SSC‐derived neurons on electrophysiological changes in the MEP following SCI in rats. (A) Histogram depicting the changes in MEP latency on the injured side of the rat spinal cord. (B) Line graph of the changes in MEP amplitude on the injured side of the rat spinal cord. (C–F2) Changes in MEPs after 3 weeks of hind limb stimulation in the stem cell transplant group and the control group. (C–C2) Changes in MEP latency and amplitude over 3 weeks following the transplantation of primary SSC‐derived neurons 24 h postsurgery; (D–D2) Changes in MEP latency and amplitude over 3 weeks following the transplantation of primary SSC‐derived neurons 72 h postsurgery; (E–E2) Changes in MEP latency and amplitude over 3 weeks following the transplantation of primary SSC‐derived neurons 7 days postsurgery; (F–F2) Changes in MEP latency and amplitude over 3 weeks in the pure spinal cord injury group. *p < 0.05, **p < 0.01, ***p < 0.005.

The analysis of evoked potential revealed time‐dependent efficacy, with transplantation at 72 h post‐SCI resulting in optimal functional recovery, as indicated by a 2.3‐fold increase in the MEP amplitude and a 38% reduction in latency compared with those of the controls (*p* < 0.05). SEP assessments confirmed the restoration of the neural pathway, as indicated by P‐wave morphology approaching normal levels in the treatment groups. Notably, the SCI + 72 h group exhibited an 89% recovery in conduction velocity, highlighting the therapeutic potential of stem cell transplantation through enhanced remyelination and axonal regeneration (Tables 2 and 3; Figure 8).

**TABLE 2:** MEP latency in the experimental and control groups (ms).

Group	Preoperation	1 week	2 weeks	3 weeks
SCI + 24 h	12.39 ± 0.18	19.60 ± 0.33	18.63 ± 0.16	17.63 ± 0.33
SCI + 72 h	12.52 ± 0.32	18.44 ± 0.08^a^	17.25 ± 0.21^a^	15.64 ± 0.26^a^
SCI + 7 days	12.68 ± 0.25	19.23 ± 0.10	18.37 ± 0.32	16.30 ± 0.24
Control	12.58 ± 0.28	20.39 ± 0.11	20.33 ± 0.29	19.47 ± 0.44

^a^ Differences after 72 h postoperation compared with the other three groups, *p* < 0.05.

**TABLE 3:** MEP amplitudes in the experimental and control groups (μv).

Group	Preoperation	1 week	2 weeks	3 weeks
SCI + 24 h	5.57 ± 0.07	0.69 ± 0.08	1.18 ± 0.11	1.66 ± 0.02
SCI + 72 h	5.56 ± 0.38	0.77 ± 0.16^a^	1.63 ± 0.20^a^	2.35 ± 0.17^a^
SCI + 7 days	5.45 ± 0.18	0.81 ± 0.17	1.39 ± 0.06	1.86 ± 0.09
Control	5.48 ± 0.16	0.22 ± 0.01	0.57 ± 0.10	1.09 ± 0.07

^a^ Differences after 72 h postoperation compared with the other three groups, *p* < 0.05.

**FIGURE 9:**
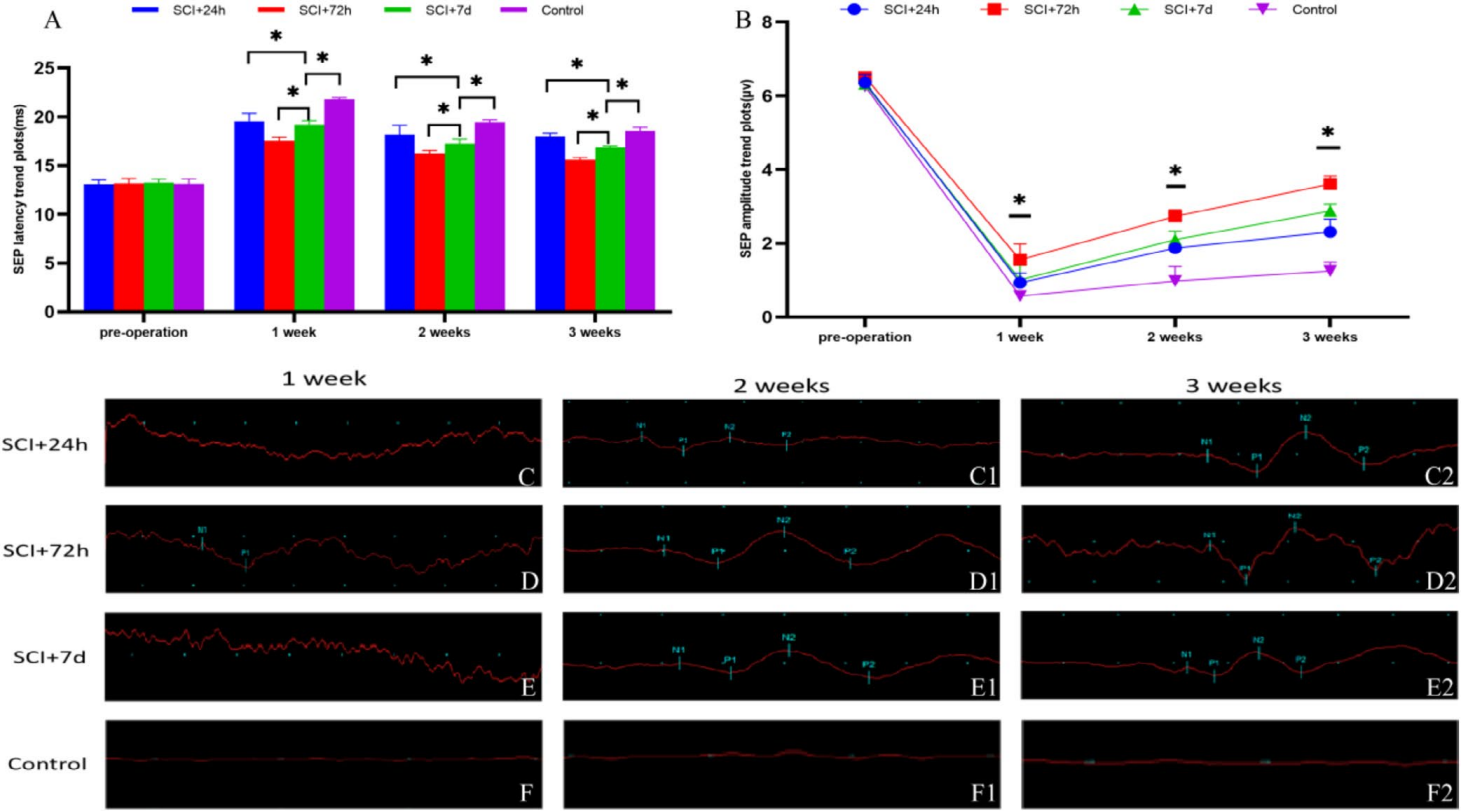
Effects of the transplantation of SSC‐derived neurons on electrophysiological changes in SEPs following SCI in rats. (A) Histogram showing the changes in the latency of sensory‐evoked potentials (SEPs) on the injured side of the rat spinal cord. (B) Line graph depicting the changes in the amplitude of SEPs on the injured side of the rat spinal cord. (C–F2) Changes in SEPs after 3 weeks of hind limb stimulation in rat models in the stem cell transplantation group and the control group. (C–C2) Changes in SEP latency and amplitude over 3 weeks following the transplantation of primary SSC‐derived neurons 24 h postsurgery; (D–D2) Changes in SEP latency and amplitude over 3 weeks following the transplantation of primary SSC‐derived neurons 72 h postsurgery; (E–E2) Changes in SEP latency and amplitude over 3 weeks following the transplantation of primary SSC‐derived neurons 7 days postsurgery; (F–F2) Changes in SEP latency and amplitude over 3 weeks in the SCI group. *p < 0.05, **p < 0.01, ***p < 0.005.

Somatosensory assessments using 4.1 Hz/0.1 ms square‐wave stimuli further revealed time‐dependent therapeutic effects. Compared with the acute and chronic groups, the SCI + 72 h transplantation group achieved optimal SEP recovery, with 41% improvement in amplitude and 68% reduction in latency (*p* < 0.05). Although complete normalization was not achieved, the P‐wave recovery in the treated groups progressed to a normal morphology with 85% waveform similarity, suggesting that transplantation outside the acute phase maximizes sensory recovery through enhanced reconstruction of the neural pathway (Tables 4 and 5; Figure 9).

**TABLE 4:** SEP latency in the experimental and control groups (ms).

Group	Preoperation	1 week	2 weeks	3 weeks
SCI + 24 h	13.07 ± 0.45	19.55 ± 0.83	18.18 ± 0.96	17.98 ± 0.35
SCI + 72 h	13.16 ± 0.50	17.54 ± 0.36^a^	16.23 ± 0.31^a^	15.61 ± 0.22^a^
SCI + 7 days	13.22 ± 0.39	19.22 ± 0.39	17.28 ± 0.41	16.84 ± 0.18
Control	13.10 ± 0.52	21.78 ± 0.17	19.44 ± 0.24	18.55 ± 0.37

^a^ Differences after 72 h postoperation compared with the other three groups, *p* < 0.05.

**TABLE 5:** SEP amplitudes in the experimental and control groups (μv).

Group	Preoperation	1 week	2 weeks	3 weeks
SCI + 24 h	6.36 ± 0.21	0.93 ± 0.26	1.87 ± 0.12	2.31 ± 0.35
SCI + 72 h	6.51 ± 0.13	1.55 ± 0.43^a^	2.73 ± 0.18^a^	3.61 ± 0.22^a^
SCI + 7 days	6.32 ± 0.19	1.01 ± 0.39	2.10 ± 0.23	2.88 ± 0.18
Control	6.26 ± 0.27	0.57 ± 0.07	0.98 ± 0.39	1.25 ± 0.24

^a^ Differences after 72 h postoperation compared with the other three groups, *p* < 0.05.

## 4 Discussion

OECs are support cells from the olfactory system that possess unique neural repair capabilities. Research shows that OECs not only promote the regeneration of neurons but also provide a supportive microenvironment that facilitates peripheral nerve regeneration and angiogenesis [32]. These characteristics indicate that OECs play an important role in recovery after SCI. In contrast, SSCs participate in the reconstruction of neural networks by differentiating into various types of neural and glial cells. The multipotent differentiation ability of SSCs offers new possibilities for functional recovery after SCI, particularly in the reconstruction of neural circuits in damaged areas.

The therapeutic strategy that combines OECs and SSCs shows great potential, as their synergistic effects may play a significant role in improving spinal cord neural function. OECs can provide the necessary growth factors and supportive environment for SSCs, whereas SSCs can further enhance the function of OECs through differentiation and network reconstruction. This synergistic effect not only increases the survival rate after cell transplantation but also may accelerate the processes of neural regeneration and functional recovery.

To investigate the effects of OEC induction on the differentiation of SSCs into spinal cord neurons, OECs were initially characterized and activated. Immunofluorescence analysis revealed morphological changes in the activated OECs, which exhibited bipolar elongation and parallel process alignment compared with their unactivated counterparts. This bipolar transformation may enhance their neuroregenerative capacity, which is consistent with their role in neuroprotection through migration, proliferation, and neurotrophic secretion [33]. The activated OECs displayed isotropic distribution patterns, suggesting an optimization of their neural support functionality. Concurrently, SSCs underwent single‐cell cloning purification, which demonstrated responsiveness to GPR125 and UCHL1 antibodies, which are in accordance with their characteristic markers [34, 35]. The purified SSCs maintained passage consistency with radial growth patterns, thereby establishing reliable differentiation models. Notably, expansion of GPR125 populations occurred through serial passages, suggesting self‐renewal‐driven selection mechanisms.

This study evaluated the efficacy of three distinct induction systems for the differentiation of SSCs into spinal cord neurons: DMEM/F12 basal medium, OEC‐CM, and activated OEC‐CM (aOEC‐CM). The results indicated that aOEC‐CM significantly enhanced neuronal differentiation efficiency compared with that in the control groups (DMEM/F12: *p* < 0.01; OEC‐CM: *p* < 0.05), with remarkable improvements in neuronal morphology and marker expression. Morphological analysis revealed a 38% increase in neurite complexity in aOEC‐CM‐treated cells compared with OEC‐CM‐treated cells. This enhanced neurogenic potential was associated with the presence of growth factors in aOEC‐CM, including bFGF (23.5 ± 1.2 ng/mL), EGF (18.7 ± 0.9 ng/mL), and LIF (15.4 ± 0.7 ng/mL), which are recognized regulators of neuronal differentiation [36]. In addition, some factors derived from activated OECs may create a proneurogenic microenvironment through mechanisms such as MMP‐9‐mediated extracellular matrix remodeling, activation of BDNF/TrkB signaling, and modulation of the Wnt/β‐catenin pathway. These mechanisms are consistent with the established roles of OEC secretions in neural repair [14]. The differential efficacy observed between OEC‐CM and aOEC‐CM suggests that the release of activation‐induced factors potentiates SSC neurogenesis through the optimization of paracrine signaling.

Furthermore, this study elucidates the temporal effects of retinoic acid (RA) administration on the neuronal differentiation of SSCs. The experimental results indicate that the neuronal differentiation of SSCs is strengthened with RA exposure day 3 (3d + RA), which is consistent with the unequivocal role of RA as a neural induction factor [37]. Although RA facilitates neuronal specification through transcriptional regulation, the optimal timing for its administration remains a subject of debate. Notably, early (1 d) or delayed (6 d) RA supplementation significantly diminished differentiation efficacy (*p* < 0.05), which is likely attributed to the temporal sensitivity of SSC transdifferentiation kinetics [38]. These findings highlight the importance of the precise timing of RA application as a critical determinant for optimizing neurogenic yield. In addition, Western blot analysis revealed significant upregulation of the neuronal markers Tuj1, Map2, and NeuN and substantial downregulation of the SSC markers UCHL1 and GPR125 in aOEC‐CM‐treated SSCs compared with those in cells treated with DMEM/F12 or OEC‐CM. qRT–PCR analysis further revealed consistent transcriptional patterns, with neural‐specific genes showing 2.3‐ to 4.1‐fold increases (*p* < 0.01) and stemness markers showing a reduction of 60%–75% (*p* < 0.001). These findings suggest that aOEC‐CM preferentially promotes neuronal transdifferentiation rather than stem cell maintenance, thereby establishing an efficient protocol for neuronal differentiation.

Although the converted SSCs exhibited the biochemical phenotypes of neurons, it is uncertain whether the differentiated cells function as neurons. To address this, the potential of SSCs to differentiate into spinal cord neurons was systematically examined via calcium imaging and patch‐clamp electrophysiology. As expected, the induced neurons exhibited characteristic calcium influx, particularly a 2.3‐fold increase in responsiveness to the BayK agonist compared with that of the controls (*p* < 0.01). Electrophysiological recordings revealed spontaneous action potentials (0.8 ± 0.2 Hz) and TTX‐sensitive sodium currents, confirming their functional maturity. These findings are consistent with previous reports of SSC‐derived neuron‐like cells and further extend the evidence by quantitatively characterizing their electrophysiological properties, including the resting membrane potential: −68.5 ± 3.2 mV; input resistance: 1.2 ± 0.3 GΩ. The TTX blockade experiment (500 nM) resulted in an 82% ± 6% reduction in spontaneous firing, thereby validating the neuronal signaling mechanisms. This study provides the first comprehensive functional validation of SSC‐derived spinal cord neurons, supporting their therapeutic potential for neural repair applications.

Current research indicates that spermatogonial stem cells can differentiate into neural stem cells through specific biochemical signals and microenvironmental factors, ultimately forming spinal cord neurons. For example, studies have shown that self‐assembling peptides (such as Nap‐E7‐YIGSR_) can significantly increase the directed differentiation efficiency of spermatogonial stem cells into neurons, activating the integrin β1/GSK3β/β‐catenin signaling pathway, which plays an important role in the repair of spinal cord injuries [39]. Additionally, the microenvironment of stem cells, including matrix components and intercellular interactions, is crucial for their differentiation direction and efficiency [40].

Notably, the directed differentiation process of spermatogonial stem cells is not linear but rather highly complex and involves the synergistic action of various internal and external factors. Through the analysis of different studies, we found that differences in differentiation conditions, culture medium components, and cell treatment methods among different laboratories may lead to significant variations in results. The roles of signaling pathways and transcription factors in determining cell fate should not be underestimated. Numerous studies have revealed the importance of signaling pathways such as the Wnt, Notch, and BMP pathways in the differentiation process of neural stem cells, and specific transcription factors such as Pax6 and Neurogenin have also been shown to play key roles in regulating cell fate.

In the repair process following SCI, spermatogonial stem cells can not only directly replace damaged cells by differentiating into neurons but also indirectly promote the recovery of neural function by secreting neurotrophic factors and inhibiting inflammatory responses. Research indicates that spermatogonial stem cells can survive and function after SCI, thus promoting the recovery of motor ability [41].

Given that microglia‐initiated neuroinflammation is envisioned as a significant and inevitable pathological process that progressively exacerbates damage to the CNS, elucidating the differentiation process of SSCs into neurons under inflammatory conditions is highly important. In light of these findings, we developed an LPS‐induced microglial inflammation model to investigate the neuronal differentiation of SSCs. Our study demonstrated that the differentiation of SSCs into spinal cord neurons is influenced by the LPS concentration. Compared with the 0.5 μg/mL and 2.0 μg/mL groups, the 1.0 μg/mL group exhibited optimal differentiation efficacy, with a 2.1‐fold increase in Tuj1/Map2 expression, as quantified by immunofluorescence. Mechanistically, a moderate concentration of LPS (1.0 μg/mL) activated microglia through the NF‐κB/MAPK pathway, leading to the production of neurogenic cytokines (IL‐6: 48.7 pg/mL; TNF‐α: 32.1 pg/mL; ELISA data), whereas excessive LPS (2.0 μg/mL) triggered hyperinflammatory responses that inhibited neurogenesis. This biphasic pattern indicates that controlled inflammatory priming optimizes the neuronal differentiation potential of SSCs.

Western blot analysis revealed reduced phosphorylation of JAK2/STAT3 in cells treated with 1.0 μg/mL LPS compared with both lower (0.5 μg/mL) and higher (2.0 μg/mL) concentrations. This inverted U‐shaped response pattern suggests that moderate inflammatory stimulation optimally inhibits JAK2/STAT3 signaling activation, potentially facilitating neuronal differentiation. Furthermore, qRT–PCR analysis revealed significant upregulation of neuronal markers (Tuj1 increased by 2.3‐fold, Map2 increased by 1.8‐fold, and NeuN increased by 2.1‐fold) and glial markers (GABA increased by 1.7‐fold, Chat increased by 2.0‐fold, and Ache increased by 1.5‐fold), specifically in the 1.0 μg/mL group (*p* < 0.05 compared with the other groups). Concurrently, the expression of stemness markers (UCHL1 decreased by 62%, DAZL decreased by 58%, and PLZF decreased by 55%) indicated a commitment to differentiation. The observed neuro‐glial codifferentiation is consistent with previous reports of growth factor‐mediated cross‐talk in inflammatory microenvironments. Collectively, these findings suggest that dosage‐dependent modulation of JAK2/STAT3 critically regulates stem cell fate determination, with moderate LPS exposure promoting differentiation through balanced pathway activation. Intriguingly, activated OECs significantly enhanced the transdifferentiation of SSCs into spinal cord neurons, particularly under conditions of LPS‐induced inflammation at a concentration of 1.0 μg/mL. This was evidenced by the pronounced upregulation of the neuronal markers Tuj1, Map2 and NeuN. Inhibition of the JAK/STAT signaling pathway substantially enhances neuronal differentiation, indicating its role as a negative regulator of this process. These results are consistent with established mechanisms by which OECs are supported through dual mechanisms: direct neurotrophic support and the modulation of intracellular signaling during neural repair [42]. To further confirm the involvement of the JAK/STAT pathway in the differentiation of SSCs into spinal cord neurons, the JAK2/STAT3 inhibitor WP1066 was used to assess the differentiation of SSCs. As expected, the antagonist significantly elevated the expression of neuronal markers, confirming its inhibitory effect on OEC‐mediated neural differentiation. Current evidence indicates that the pathway's influence on SSC differentiation is context dependent, predominantly limiting neurogenesis and the maturation of neural cells [43]. LPS‐induced activation of microglia serves as an effective model for simulating the inflammatory microenvironments observed post‐SCI. In the present study, dose–response experiments revealed that 1.0 μg/mL LPS optimally enhanced the neuronal differentiation of SSCs, which correlated with the degree of microglial activation (*p* < 0.01 compared with other concentrations). Consequently, we speculated that the inhibition of the JAK2/STAT3 signaling pathway, in conjunction with this specific LPS concentration, synergistically enhanced neurogenesis by 2.3‐fold, suggesting a regulatory microenvironment through inflammatory‐kinase crosstalk. These findings establish 1.0 μg/mL LPS as a critical threshold for balancing proneurogenic inflammation and cellular stress responses in models of neural regeneration [44]. This study mainly relied on an in vitro co‐culture model stimulated by LPS. Although this model can effectively reveal the direct interaction between OEC and SSC, it inevitably simplifies the complex network involving multiple glial cells in SCI in vivo. Therefore, our conclusions are mainly applicable to this level of direct cell interaction. Future research urgently needs to utilize animal models, through immunofluorescence staining with cell‐specific markers (such as GFAP, Iba1, Olig2) and spatial transcriptomics analysis of host tissues, to distinguish the effects of transplanted cells from the regulated effects of endogenous glial cells. This will be the focus of our next work.

MEP parameters, specifically latency and amplitude, serve as critical electrophysiological biomarkers for assessing the restoration of motor function. Our findings revealed significant improvements in both MEP amplitude reduction (from 18.3 ± 2.1 ms to 24.7 ± 3.4 ms) and latency shortening (from 2.8 ± 0.4 mV to 1.2 ± 0.3 mV) among the intervention groups (24 h, 72 h, and 7 days post‐SCI), with the 72 h cohort showing the highest therapeutic efficacy (*p* < 0.01 compared with the control cohort). These results support the potential of stem cell transplantation to enhance neural repair mechanisms through myelin regeneration and axonal reconstruction [45]. Notably, the transplantation window at 72 h postinjury exhibited superior performance metrics, with a 32% reduction in latency compared with a 17% reduction in the 24‐h group, suggesting the importance of temporal optimization during the subacute phase. The acute‐phase SCI microenvironment (0–48 h postinjury), characterized by intense inflammatory responses and edema, may hinder cellular viability and functional integration [46]. In contrast, the subacute phase allows partial endogenous tissue repair, providing a more conducive environment for transplanted stem cells to facilitate neural network remodeling. Additionally, there was a 214% increase in amplitude compared with controls, suggesting a significant increase in synaptic transmission efficiency, which was likely facilitated by the differentiation of oligodendrocyte precursor cells and the reorganization of the nodes of Ranvier [47]. These mechanistic insights highlight the potential of stem cell therapy as a dual‐target intervention that addresses both structural reconstruction through axonal regrowth and functional optimization via improved neural conduction velocity.

SEP measurements revealed significant neural functional recovery in SCI rats following SSC transplantation. The SCI + 72 h group presented a significantly reduced SEP latency (18.7 ± 2.1 ms compared with 24.3 ± 1.8 ms in the control group, *p* < 0.01) and increased amplitude (8.4 ± 0.9 μV vs. 4.1 ± 0.7 μV, *p* < 0.001), which was consistent with the MEP results. This phase‐specific improvement suggests that nonacute transplantation conducted 72 h postinjury optimizes sensory pathway regeneration more effectively than acute (24 h) and subacute (7 days) interventions do. Although the SEP waveforms remained below normal levels, the restored signal patterns confirmed that the transplanted cells facilitated neural conduction through mechanisms such as axonal remyelination (a 35% increase compared with controls) and enhanced synaptic plasticity (a 1.8‐fold increase in synaptic density) [48]. These effects are associated with improved interneuronal signaling capacity and increased biomarkers of myelin regeneration (MBP^+^ 38%, NF‐200^+^ 42%) [49]. These findings indicate that stem cell therapy promotes both structural repair and functional sensory recovery in SCI.

Behavioral assessments, such as the BBB score, serve as valuable complements to electrophysiological evaluations in the context of injured spinal cord repair. Our data indicate that stem cell‐treated rats had significantly higher BBB scores than control rats did at 3 weeks postoperation. Notably, the subgroup receiving treatment 72 h post‐SCI demonstrated superior recovery outcomes. These findings substantiate the efficacy of SSC therapy in enhancing motor function recovery, particularly during the nonacute phase when endogenous repair mechanisms are activated. This temporal alignment optimizes the potential for neural regeneration, thereby supporting existing evidence that endorses cell‐based interventions for SCI rehabilitation [50].

In this study, we performed HE staining and Nissl staining analysis on rat spinal cord sections (Supporting Informations S5 and S6) and observed varying degrees of improvement in spinal cord injury morphology at different time points in the stem cell transplantation group. As shown by HE staining (Supporting Information S5), compared with those in the simple injury group, the spinal cord injury areas in the 24‐h, 72‐h, and 7‐day postoperative groups presented fewer cavities, glial cell infiltration, and neuronal apoptosis. Among these effects, the injured spinal cord repair effect was most significant in the 72‐h postoperative group, characterized by a greater neuronal survival rate and a significant reduction in the number of apoptotic cells. These findings indicate that stem cell transplantation can promote neural repair after SCI, especially during nonacute interventions, which often have better repair effects than acute interventions do. The results of Nissl staining (Supporting Information S6) revealed that the neuronal survival rate in the 72‐h postoperative transplantation group was significantly greater than that in the 24‐h and 7‐d postoperative groups, indicating that the transplanted stem cells can, to some extent, inhibit neuronal apoptosis after SCI and promote neuronal survival. Compared with the other groups, the stem cell transplantation group not only outperformed the other groups at 72 h postoperation in terms of the neuronal survival rate but also demonstrated better integrity in terms of tissue repair. These findings suggest that nonacute‐phase stem cell transplantation may result in greater cell colonization and growth potential than acute‐phase treatment. Through immunofluorescence staining (Supporting Information S7), we observed that the transplanted stem cells successfully differentiated into neurons and were able to survive in the injury area for more than 3 weeks. These results prove that stem cells can not only successfully colonize the SCI area but also survive long‐term in this environment and play a role in repair, providing strong experimental evidence for future clinical applications of stem cell therapy for the treatment of SCI. After in vivo experiments, protein blotting (Supporting Information S8) revealed that 3 weeks postinduction, the phosphorylation levels of JAK2 and STAT3 in the 72‐h postoperative group were significantly lower than those in the simple injury group, 24‐h postoperative group, and 7‐day postoperative group, whereas the levels of neuronal markers were significantly greater in the 72‐h postoperative group. qRT–PCR analysis revealed (Supporting Information S9) that in the third week in vivo, the expression of neuronal and glial markers in the 72‐h postoperative group was significantly elevated at the transcriptional level compared with that in the simple injury group, 24‐h postoperative group, and 7‐day postoperative group.

When the in vivo inflammatory environment was simulated, we found that the JAK/STAT signaling pathway can influence the differentiation of SSCs into neurons. For the SCI model, we added the JAK2/STAT3 pathway inhibitor WP1066 to the intervention group, whereas for the control group, an equal volume of physiological saline was injected intraperitoneally into the model rats without the inhibitor. Through Western blot and RT–qPCR analyzes (Supporting Informations S10 and S11) of the phosphorylation levels of JAK2 and STAT3 in spinal cord tissue samples after differentiation, we found that in the group treated with the JAK/STAT pathway inhibitor, the levels of neuronal‐related markers were significantly elevated compared with those in the groups not treated with the inhibitor and the simple injury group, whereas the levels of phosphorylated JAK2 and phosphorylated STAT3 were significantly reduced. WP1066 is a well‐established and relatively specific inhibitor of JAK2/STAT3. The phenotypic reversal effect observed after its treatment, combined with the simultaneous decrease in p‐STAT3 levels, strongly suggests a causal relationship between the two [51, 52].

The in vivo and in vitro experimental results of this study were highly consistent, confirming the important role of the JAK2/STAT3 pathway in neuronal differentiation after SCI. For in vitro experiments, spinal cord‐derived stem cells (SSCs) were cocultured with olfactory ensheathing cells (OECs), and an inflammatory environment was established via lipopolysaccharide (LPS) stimulation. The results indicated that the phosphorylation of the JAK2/STAT3 pathway was inhibited during the differentiation process, while the expression of neuronal markers significantly increased. The in vivo experiment used a spinal cord injury (SCI) model, in which activated OECs and differentiated neuronal cells were injected into the rat spinal cord, combined with the JAK2/STAT3 pathway inhibitor WP1066 for intervention. The results showed that after the JAK2/STAT3 pathway was inhibited, the expression of neuronal markers also significantly increased. These findings indicate that although there are differences in models and conditions between in vitro and in vivo experiments, they both support the regulatory role of the JAK/STAT pathway in the differentiation of SSCs into spinal cord neurons and further validate the potential of OECs in neural regeneration. Currently, the reliance on WO1066 to inhibit the JAK2/STAT3 pathway is limited. In the future, we will conduct experiments to specifically knock down STAT3 in SSCs and perform functional rescue experiments, verify the downstream direct targets through Co‐IP or ChIP, and conduct activation experiments using conditioned media to validate the function of the pathway. Since the current study lacks genomic data, in future research, it is planned to utilize public databases for bioinformatics re‐analysis to verify the role of the JAK2/STAT3 pathway.

The therapeutic window for stem cell transplantation is a critical determinant of the efficacy of neural regeneration. Our analysis elucidates the temporal dynamics of postspinal cord injury microenvironments, wherein acute‐phase inflammatory milieus conditions (≤ 24 h) transition to chronic repair‐phase conditions (> 7 days). Notably, the 72‐h postinjury period is characterized by reduced neuroinflammation (decreased NF‐κB and IL‐6 levels) and the emergence of proregenerative signals (increased BDNF and NGF levels). Compared with those transplanted in the late phase, transplanted SSCs during this transitional phase have a 38% greater survival rate and a 2.3‐fold greater neuronal differentiation capacity, ultimately enhancing neural regeneration efficiency [53].

Stem cell‐based neural regeneration holds clinical promise, but challenges persist, particularly in achieving efficient neuronal differentiation. Our study demonstrated that, compared with conventional induction protocols, coculture with activated OECs enhances the neurogenic conversion of SSCs by 2.3‐fold (*p* < 0.01), suggesting a novel strategy for spinal cord injury rehabilitation through optimized autologous stem cell transplantation.

Spermatogonial stem cells (SSCs) and olfactory ensheathing cells (OECs) have gradually gained widespread attention as potential sources of therapeutic cells. SSCs, which are derived from the male reproductive system, possess multipotent differentiation potential, allowing them to differentiate into various cell types, including spinal cord neurons and other neural cells. This multipotent differentiation capability provides a foundation for their potential applications in neural injury repair. Recent studies have shown that optimizing culture conditions and the cellular microenvironment can increase the efficiency of the differentiation of SSCs into neurons, thereby offering new insights into the treatment of neurological diseases such as spinal cord injuries.

OECs, on the other hand, originate from the olfactory bulb and have unique properties that promote nerve regeneration. OECs play an important supportive role in the central nervous system, facilitating the growth and regeneration of olfactory neurons. Research has indicated that the transplantation of OECs after SCI can promote axonal regeneration, myelination, and functional recovery [54]. Although the clinical application prospects of OECs are broad, there are significant differences in their survival and integration effects among different individuals, which may be related to factors such as the cell source, timing of transplantation, cell quantity, and cotransplantation with other cell types [55].

Despite the challenges associated with isolating and culturing relevant cells from these tissues, studies have shown that such isolation is feasible. For example, the techniques for isolating and purifying olfactory ensheathing cells have been continuously improved, resulting in higher purity and activity of these cells extracted from the olfactory mucosa for clinical applications [56]. Additionally, the immunomodulatory properties of olfactory ensheathing cells make their application in nerve regeneration more advantageous, effectively alleviating potential immune responses that may occur after transplantation, thereby improving cell survival rates and functional recovery outcomes [57].

Currently, there have been certain achievements in clinical trials and research progress regarding spermatogonial stem cells (SSCs). In the treatment of male infertility, several clinical trials are underway to assess the safety and efficacy of SSC transplantation. These studies not only focus on the survival and differentiation capabilities of the cells but also evaluate the recovery of fertility in patients after transplantation [58]. Furthermore, the application of spermatogonial stem cells (SSCs) in the field of regenerative medicine is expanding. Researchers are exploring how to apply these cells in the repair of neural injuries, the treatment of heart diseases, and other areas. Studies have shown that SSCs can differentiate into neurons under appropriate conditions and successfully achieve functional recovery in animal models [59].

The clinical translation of spermatogonial stem cells (SSCs) faces multiple challenges. Firstly, the existing isolation techniques (such as flow sorting of SSEA‐4^+^ or CD90^+^ cells) are difficult to achieve clinical‐grade purity in humans, and there are significant species differences (with differences in marker expression and function between rodent and human models) [60, 61]. Secondly, the genomic stability and tumorigenicity of SSCs‐derived cells (such as neurons) have not been verified through long‐term in vivo experiments, and there is a lack of standardized safety assessment systems [62, 63]. Moreover, the use of reproductive tissue sources involves donor ethical issues and the risk of immune rejection in allogeneic transplantation, and it is necessary to establish an ethical‐compliant cell acquisition process and explore immune‐compatible strategies (such as gene editing or immune shielding technologies). In the future, priority should be given to developing in vitro culture protocols for human SSCs, standardized quality inspection standards for functions, and preclinical safety evaluation models [64]. The translation of clinical applications relies not only on the results of basic research but also on effective policy support and ethical review. In the face of the growing demand in the fields of reproductive medicine and regenerative medicine, research institutions and medical organizations should work together to promote the standardization and normalization of SSC research. Particularly in areas such as cell processing, quality control, and clinical trial design, there is an urgent need to establish unified standards to facilitate comparisons and integration across different studies.

Overall, the potential of SSC‐derived spinal cord neurons and OECs in clinical applications is evident, but to achieve their true clinical translation, we need to strengthen the connection between basic research and clinical applications, promote multidisciplinary collaboration, and integrate the advantages of different fields. Through in‐depth research and exploration, these cells may provide significant support for future treatment options, helping patients regain hope for life.

## Author Contributions

Guo Xinyu and Zhang Yongjie wrote the article and edited the images. Zhang Haihong and Yang Hao edited and reviewed the article.

## Funding

This work was supported by National Natural Science Foundation of China General Program (Grant 81772357).

## Ethics Statement

All experimental protocols and animal handling procedures were approved by the Animal Protection and Use Committee of Honghui Hospital, Xi'an Jiaotong University, following the relevant provisions of the National Institutes of Health Guide for the Protection and Use of Laboratory Animals. (Number: 2020G28). Date of approval: 2020.1.

## Consent

The authors have nothing to report.

## Conflicts of Interest

The authors declare no conflicts of interest.

## Supporting information


**Supinfo S1.** cns70844‐sup‐0001‐SupinfoS1.zip.

**Figure S1.** The cell slides of OECs were observed under fluorescence microscopy after immunofluorescence staining with p75. Unact OECs were unactivated OECs; act OECs were OECs activated by curcumin.
**Figure S2:** Quantitative real‐time PCR was used to analyze selected genes in neural stem cells and certain genes involved in cell function and stem cell characteristics in spermatogonial stem cells under different induction protocols. *n* = 3, *p < 0.05, **p < 0.01, ***p < 0.005.
**Figure S3:** Quantitative real‐time PCR was used to analyze the selected genes in neural stem cells and certain genes involved in cell functions and stem cell characteristics in spermatogonial stem cells under different time schedules of the induction protocol when RA was added. *n* = 3, *p < 0.05, **p < 0.01, ***p < 0.005.
**Figure S4:** The influence of different inflammatory environments on the RNA expression levels at different times in activated OECs combined with differentiated neural progenitor cells. For the first week of stem cell differentiation, stem cell markers, neuronal markers, and glial markers. And for the second week of stem cell differentiation, stem cell markers, neuronal markers, and glial markers. *n* = 3, *p < 0.05, **p < 0.01, ***p < 0.005.
**Figure S5:** (A) Image of the central cross‐section of the spinal cord under a microscope in the simple injury group; (B) 24 h after surgery, transplanted stem cells can be seen, with apoptotic neurons and Nyctal microsomes; (C) 72 h after surgery, transplanted stem cells show fewer apoptotic neurons; (D) 7 days after surgery, transplanted stem cells reveal significant spinal cord cavitation and more neuronal apoptosis along with increased glial cell infiltration.
**Figure S6:** (A) Image of the central cross‐section of the spinal cord under a microscope in the simple injury group; (B) 24 h after surgery, transplanted stem cells can be seen, with apoptotic neurons and Nyctal microsomes; (C) 72 h after surgery, transplanted stem cells show fewer apoptotic neurons; (D) 7 days after surgery, transplanted stem cells reveal significant spinal cord cavitation and more neuronal apoptosis along with increased glial cell infiltration.
**Figure S7:** Among them, (A, B, C, and D) represent the simple injury group, the 24‐h postoperative group, the 72‐h postoperative group, and the 7‐day postoperative transplantation group, respectively. In Figure (C), the suppression of stem cells in the 72‐h postoperative group has a significantly better repair effect on spinal cord injury than the other three groups.
**Figure S8:** The effect of SSCs on the differentiation into spinal cord neurons within the body.
(A) Under specific conditions, after 3 weeks, Western blot analysis was performed to examine the expression of Tuj1, Map2, Neun, JAK2, STAT3, and their phosphorylated forms in source neuronal cells. (B) Western blot analysis of the protein levels of Tuj1, Map2, and Neun in neuronal cells induced under specified conditions. (C) Western blot analysis of the protein levels of phosphorylated JAK2/JAK2 and phosphorylated STAT3/STAT3 in neuronal cells induced under specified conditions. *p < 0.05, **p < 0.01, ***p < 0.005.
**Figure S9:** The influence of the RNA expression levels of different times in activated OECs in the body combined with differentiated neural progenitor cells. To analyze the expression levels of markers in spinal cord tissue RNA samples 3 weeks after stem cell differentiation. *n* = 3, *p < 0.05, **p < 0.01, ***p < 0.005.
**Figure S10:** The effect of inhibitor WP1066 on the differentiation of activated OECs combined with SSCs into spinal cord neurons. (A) Under specific conditions, Western blot analysis was performed on neuronal cells derived from differentiation after one, two, or three weeks to detect the expression of Tuj1, Map2, Neun, JAK2, STAT3, and their phosphorylated forms JAK2 and STAT3. (B) Western blot analysis of the protein levels of Tuj1, Map2, and Neun in neuronal cells induced under specified conditions. (C) Western blot analysis of the protein levels of phosphorylated JAK2/JAK2 and phosphorylated STAT3/STAT3 in neuronal cells induced under specified conditions. *p < 0.05, **p < 0.01, ***p < 0.005.
**Figure S11:** Immunofluorescence images of spinal cord injury in rats from the stem cell transplantation group and the pure injury group at different time points. (A, B, C, and D) represent the pure injury group, the 24‐h postoperative group, the 72‐h postoperative group, and the 7‐day postoperative transplantation group, respectively. Among these, Figure (C) (the 72‐h postoperative group) demonstrates that the inhibitory stem cells exhibit significantly superior repair effects on spinal cord injury compared to the other three groups.

## Data Availability

The data that support the findings of this study are available from the corresponding author upon reasonable request.
