# Supplementary material for: An Optimized Conversion of Spermatogonial Stem Cells Into Spinal Cord Neurons Enhances Functional Recovery in Rats After Spinal Cord Injury
Source: CNS Neurosci Ther. 2026 Apr 15;32(4):e70844. doi: 10.1002/cns.70844 (PMC13080337; doi:10.1002/cns.70844)
Supplement: Supplementary file 2 — Figure S1. The cell slides of OECs were observed under fluorescence microscopy after immunofluorescence staining with p75. Unact OECs were unactivated OECs; act OECs were OECs activated by curcumin. Figure S2: Quantitative real‐time PCR was used to analyze selected genes in neural stem cells and certain genes involved in cell function and stem cell characteristics in spermatogonial stem cells under different induction protocols. n = 3, *p < 0.05, **p < 0.01, ***p < 0.005. Figure S3: Quantitative real‐time PCR was used to analyze the selected genes in neural stem cells and certain genes involved in cell functions and stem cell characteristics in spermatogonial stem cells under different time schedules of the induction protocol when RA was added. n = 3, *p < 0.05, **p < 0.01, ***p < 0.005. Figure S4: The influence of different inflammatory environments on the RNA expression levels at different times in activated OECs combined with differentiated neural progenitor cells. For the first week of stem cell differentiation, stem cell markers, neuronal markers, and glial markers. And for the second week of stem cell differentiation, stem cell markers, neuronal markers, and glial markers. n = 3, *p < 0.05, **p < 0.01, ***p < 0.005. Figure S5: (A) Image of the central cross‐section of the spinal cord under a microscope in the simple injury group; (B) 24 h after surgery, transplanted stem cells can be seen, with apoptotic neurons and Nyctal microsomes; (C) 72 h after surgery, transplanted stem cells show fewer apoptotic neurons; (D) 7 days after surgery, transplanted stem cells reveal significant spinal cord cavitation and more neuronal apoptosis along with increased glial cell infiltration. Figure S6: (A) Image of the central cross‐section of the spinal cord under a microscope in the simple injury group; (B) 24 h after surgery, transplanted stem cells can be seen, with apoptotic neurons and Nyctal microsomes; (C) 72 h after surgery, transplanted stem cells show fewer apoptotic [file CNS-32-e70844-s001.zip › Supplementary Materials 1/S1 OEC.doc]

The cell slides of OECs were observed under fluorescence microscopy after immunofluorescence staining with p75. Unact OECs were unactivated OECs; act OECs were OECs activated by curcumin.
